# Drivers of population divergence and species differentiation in a recent group of indigenous orchids (*Vanilla* spp.) in Madagascar

**DOI:** 10.1002/ece3.7224

**Published:** 2021-02-24

**Authors:** Cathucia F. Andriamihaja, Aro V. Ramarosandratana, Michel Grisoni, Vololoniaina H. Jeannoda, Pascale Besse

**Affiliations:** ^1^ Université de la Réunion UMR PVBMT St Pierre France; ^2^ Department of Plant Biology and Ecology University of Antananarivo Antananarivo Madagascar; ^3^ CIRAD UMR PVBMT Toamasina Madagascar

**Keywords:** ecological distances, flower traits, geographic distances, leafless, microsatellite, speciation, *Vanilla*

## Abstract

With over 25,000 species, orchids are among families with remarkable high rate of diversification. Since Darwin's time, major advances attributed the exceptional diversity of orchids to plant–pollinator interactions. However, unraveling the processes and factors that determine the phenotypic and genotypic variation of natural orchid populations remains a challenge. Here, we assessed genetic population structure and floral differentiation in recently diverged leafless *Vanilla* species in a world biodiversity hotspot, Madagascar, using seven microsatellite loci and 26 morphometric variables. Additionally, analyses were performed to test for the occurrence of any patterns of isolation by distance, isolation by environment, and isolation by adaptation and to detect possible physical barriers that might have caused genetic discontinuities between populations. Positive inbreeding coefficients detected in 22 populations were probably due to the presence of null alleles, geitonogamy and/or some admixture (sympatric species). In contrast, the only high‐altitude population showed an important rate of clonality leading to heterozygote excess. Genetic diversity was maximum in western populations, suggesting a postglacial colonization to the north and south. Clustering analyses identified seven genetic groups characterized by specific floral traits that matched five botanical descriptions in the literature. A contribution of montane refugia and river barriers on population differentiation was detected. We also detected combined effects of IBD/IBE and IBE/IBA on genetic differentiation and suggested this pattern is more likely determined by ecological isolation, although pollinator‐mediated divergent selection could not be ruled out for some of the species. Overall, this study provides further insights on speciation in orchids, a group for which Madagascar shows one of the world's highest level of endemism and confirms the importance of the peculiar biogeography of the island in shaping species differentiation.

## Graphical abstract

The aim of our study was to resolve evolutionary processes in a group of recently diverged Vanilla species in Madagascar, using a population genetics approach, with a combination of molecular data (microsatellites), floral morphological data, ecological data, and geographic distances. Clustering analyses identified seven genetic groups characterized by specific floral traits that matched five botanical descriptions in the literature. A contribution of montane refugia and river barriers on population differentiation was detected. We also detected combined effects of IBD/IBE and IBE/IBA on genetic differentiation and suggested this pattern is more likely determined by ecological isolation, although pollinator‐mediated divergent selection could not be ruled out for some of the species.

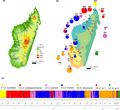

## 1 INTRODUCTION

Understanding how and at what rate phenotypic and genetic differentiation occurs across natural populations is one of the most fundamental questions in evolutionary biology (Ghalambor et al., 2007; Lekberg et al., 2012; Schmid & Guillaume, 2017). It is now well established that three mechanisms and their interactions shape the patterns of population diversity and structure, which are reduced gene flow, genetic drift, and natural selection (Gandon & Nuismer, 2009; Johnson et al., 2018; Wright, 1931). They are strongly influenced by many interrelating factors including historical events, human activities, distance and barriers, and ecological variations (Gomez et al., 2009; Wright, 1943; Zhang et al., 2014). However, the last two factors were identified as the most important ones (Mallet et al., 2014; Noguerales et al., 2016; Rundle & Nosil, 2005; Wright, 1931, 1943). First, geographic barriers can affect evolutionary processes by interrupting gene flow and therefore increasing population divergence (Capparella, 1987; Sobel, 2016). Nevertheless, in the absence of barriers, genetic differentiation can still occur. The pattern of isolation by distance (IBD) suggests a decrease of gene flow between populations as distance increases, triggering genetic differentiation via genetic drift and/or selection (Frankham et al., 2010; Ramírez‐Barrera et al., 2019; Wright, 1943). On the other hand, selection is the primary factor for divergence in isolation by ecology or ecological speciation *lato* sensu (Orsini et al., 2013; Shafer & Wolf, 2013; Wang & Bradburd, 2014): In this case, gene flow between populations is reduced due to local adaptation to divergent environments (Shafer & Wolf, 2013).

Past climatic oscillations have been argued to be the major factors affecting current distributions and diversity of many plant species (Gomez et al., 2009; Médail & Diadema, 2009; Zhang et al., 2014). Climatic variations can threaten several plant species and reduce plant community diversity, as shown by the extinction of European tree species during the Plio‐Pleistocene (Svenning, 2003). However, they can also be an important force driving genetic differentiation and speciation (Hewitt, 1996; Vences et al., 2009). For example, climatic variations along latitudinal gradients may affect organism functional traits such as phenology (Olsson & Ågren, 2002), physiology (Bognounou et al., 2010; De Frenne et al., 2012), and morphology (Feijó et al., 2019; Olsson & Ågren, 2002; Winn & Gross, 1993) and lead to plastic or genotypic shifts (Byars et al., 2007). Correlation analysis of neutral genetic variations versus geographic distances allows testing for IBD. On the other hand, a correlation between genetic divergence and morphological divergence can reveal isolation by adaptation (IBA) where barriers to gene flow between populations from different habitats arise following adaptive trait divergence (Nosil et al., 2008; Shafer & Wolf, 2013; Wang & Bradburd, 2014). When adaptive traits cannot be targeted, various cofactors may be used to test for isolation by ecology (Ramírez‐Barrera et al., 2019; Shafer & Wolf, 2013; Wang & Bradburd, 2014). For this purpose, environmental data (excluding geographic data) may serve as a proxy in correlation analysis with neutral genetic data to assess isolation by environment (IBE). IBE is caused by the reduction of exchanges between populations from ecologically divergent habitats due, for example, to the low recruitment in local environment. The roles of spatial isolation‐by‐distance and isolation‐by‐ecology patterns are complex in population differentiation and speciation processes, as they can be highly correlated and often act together as isolation mechanisms. Evolutionary patterns can be quite complex in plants (Lowry et al., 2008; Waser & Campbell, 2004), particularly in Orchidaceae. Indeed, orchid species evolve occasionally in sympatry (Barone Lumaga et al., 2012; Nielsen & Siegismund, 1999; Pansarin & Ferreira, 2019), produce numerous seeds with long‐range dispersal ability (Arditti & Ghani, 2000), can reproduce clonally (Batygina et al., 2003), and hybridize (Arduino et al., 1996; Nielsen & Siegismund, 1999). Potential ecological drivers of orchid diversification are multiple, such as adaptation to pollinators (Ayasse et al., 2011; Dressler, 1982; Van der Niet et al., 2014), mycorrhizal specificity (Otero et al., 2007), differences in flowering phenology, and floral characteristics (color, form and fragrance) resulting from adaptation to different environmental conditions (Givnish et al., 2015; Pansarin & Ferreira, 2019; Sun et al., 2015) and leading to isolation by ecology. Although the various patterns namely IBD, IBE, and IBA have gained attention in the literature (e.g., Garot et al., 2019; Liu et al., 2018; Noguerales et al., 2016; Orsini et al., 2013; Shafer & Wolf, 2013), few studies have evaluated their joint contribution in orchid species differentiation (e.g., Jaros et al., 2016; Mallet et al., 2014), and no research efforts, to our knowledge, have been invested so far on this subject regarding *Vanilla* species divergence.

The *Vanilla* Plumier ex Miller (34.6 Ma, Bouetard et al., 2010) belongs to the Orchidaceae, one of the most diverse family of angiosperms (Bouetard et al., 2010; Gigant et al., 2011). It comprises about 132 species (Andriamihaja et al., 2020) distributed throughout tropical forests between the 27th north and south parallels, but are absent in Australia (Gigant et al., 2011; Portères, 1954). With nine wild *Vanilla* species described, the Southwest Indian Ocean (SWIO) region ranks fourth among the richest regions and first in terms of leafless species, most being located in Madagascar (Gigant et al., 2011; Portères, 1954). Leaflessness has evolved independently at least three times in the *Vanilla* genus (Bouetard et al., 2010). Within the SWIO region, seven leafless species can be found, ensuing from the adaptation of an African leafy ancestor to xeric conditions (Bouetard et al., 2010). As they are part of a recent monophyletic group (4.4 Ma) (Bouetard et al., 2010), these species retain numerous similarities in their morphology (Allorge‐Boiteau, 2013; Andriamihaja et al., 2020; Cribb & Hermans, 2009; Portères, 1954; Soto‐Arenas & Cribb, 2010), making their identification difficult. Chloroplast DNA phylogenetic analyses also failed to distinguish the various species from this group (Bouetard et al., 2010). Such recent and closely related species have always been recognized to be an interesting model for understanding speciation processes (Lowry, Modliszewski, et al., 2008; Van der Niet et al., 2014).

Our aim is to unravel wild leafless *Vanilla* species diversification processes in Madagascar. The island of Madagascar is a perfect place for studying evolution, thanks to a high level of endemism attributed to a large heterogeneity of habitats, a small spatial scale, and a long history of geographic isolation (Vences et al., 2009; Warren et al., 2015; Wilmé et al., 2006). Actually, the level of endemism is more than 80% among plant species (Callmander et al., 2011; Goodman & Benstead, 2005; Phillipson et al., 2006) and even higher for animal taxa, ranging from 52% in birds to 100% in amphibians and lemurs (Goodman & Benstead, 2005). Habitat diversity is expressed by different biomes with unique vegetation composition and sharp borders, for instance, the long central highland plateau that rises along a latitudinal axis and separates west and east bioclimatic regions (Vences et al., 2009; Wilmé et al., 2006). Madagascar hosts more than 1,000 orchid species (Cribb & Hermans, 2007) including five leafless and two leafy *Vanilla* species (Allorge‐Boiteau, 2005, 2013; Cribb & Hermans, 2009; Portères, 1954). Over 90% of Malagasy orchids are endemic to the island (Cribb & Hermans, 2007). In this study, we generated ecological, genotypic (using variable microsatellite markers), and morphological data for leafless *Vanilla* populations from Madagascar. We determined the genetic, morphological structure and differentiation of populations, to identify possible geographic barriers and to quantify the effects of isolation by distance (IBD), isolation by adaptation (IBA), and isolation by environment (IBE) in diversification processes.

## 2 MATERIALS AND METHODS

### 2.1 Study site

**FIGURE 1:**
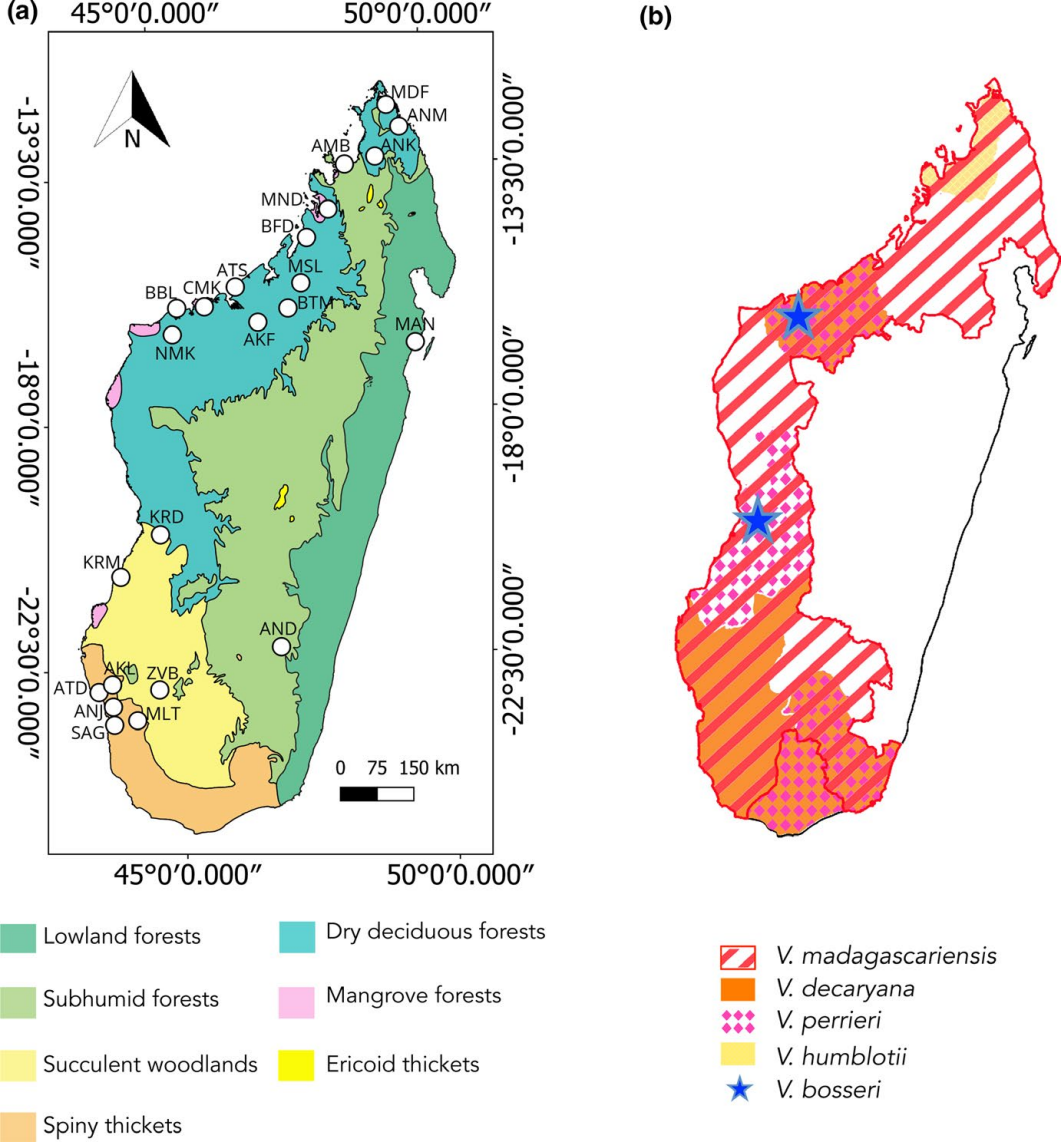
Map of studied populations, habitat types, and distribution of leafless *Vanilla* species in Madagascar. (a) Map shows the seven ecoregions of Madagascar based on WWF’s ecoregions in Burgess et al. (2004). Circles indicate geographic positions of the 23 localities with *Vanilla* populations. (b) Map represents the distribution area of the five known leafless *Vanilla* species in Madagascar according to Portères (1954), Cribb and Hermans (2009) and Allorge‐Boiteau (2013)

Fieldworks were performed in 23 sites (Table S1, Figure 1) within the distribution range of Malagasy leafless *Vanilla* species (Figure 1). Madagascar is located in the Indian ocean, southeast of Africa, around 20°S and 47°E, in the intertropical zone. A rugged chain of mountains separates the island from north to south, into dry forests (in the west) and rain forests (in the east) (Waeber et al., 2015). Several factors shaped the island climatic conditions such as topography, landform, maritime influence, and prevailing wind conditions (Cornet, 1973). During the dry season from May to October, the “Alizé” brings humid air masses from the Indian Ocean to the east coast causing precipitations (Cornet, 1973). These foggy masses move inland and drizzle in the elevated areas of the central domain. The winds become hot and dry beyond the mountains in the west, causing an accentuated dry season. During the wet season from November to April, on the other hand, the “Mousson” brings heavy rains over most of the island (Cornet, 1973). As a result, Madagascar is ecologically heterogeneous and displays five bioclimatic regions: dry, subhumid, humid, montane, and subarid (Vences et al., 2009). These ecoregions host seven typical vegetation formations: spiny thicket, succulent woodland, dry deciduous forest, subhumid forest, lowland (humid) forest, ericoid thicket, and mangrove forest (Figure 1a) (Burgess et al., 2004).

### 2.2 Study species

*Vanilla* plants are long‐lived semi‐epiphytic lianas with both vegetative and sexual reproduction (Gigant et al., 2011). Stems are thick and fleshy reaching in some cases 30 m in length, with roots growing at nodes. They generally bloom once a year, and inflorescences are in form of clusters of ephemeral flowers with a large labellum (Bosser & Lecoufle, 2011; Portères, 1954). In this study, we focused on five indigenous Malagasy *Vanilla* species (Figure 1b), among the seven botanically described leafless species in the SWIO region comprising Madagascar, Seychelles, Comoros, Mayotte and the eastern Africa coast and islands (Allorge‐Boiteau, 2005, 2013; Portères, 1954). Because of similarities regarding flower morphological traits, they are grouped within "*V*. *phalaenopsis*" by Soto‐Arenas and Cribb (2010). Populations belonging to this group grow along the Malagasy west coast where the forests are dry or spiny (spiny thickets, dry deciduous forests, succulent woodlands) except *V. madagascariensis* Rolfe also found in the subhumid forests and humid northeastern forests (Figure 1b) (Bosser & Lecoufle, 2011; Cribb & Hermans, 2009; Portères, 1954).

### 2.3 Population sampling

Between 2017 and 2020, 600 individuals from 22 populations were collected in Madagascar (Figure 1a). We used available occurrence data in the literature, including GBIF and Tropicos websites, but we have also prospected by interviewing local field botanists who are familiar with these species to choose these localities (Table S1). Fieldworks were done after a research permit (N° 201/17/MEEF/SG/DGF/DSAP/SCB.Re) was issued by the Ministry of environment and sustainable development granting an authorization to collect and export plant materials. Samples were cut from young stems from individuals at least 1 m apart, preserved in silica gel, and then lyophilized until DNA extraction. One living specimen per population was also stored in the *Vanilla* collection of the Department of Plant Biology and Ecology (University of Antananarivo). Eleven additional samples from the Manompana forest (MAN, Figure 1a) in the northeast were taken from the Biological Resource Center (BRC) Vatel collection in Reunion Island. In total, 611 individuals from 23 sites in Madagascar were studied (Figure 1a).

### 2.4 Microsatellite genotyping

DNA was extracted using the DNEASY**^®^** plant kit from Qiagen. The 611 specimens were registered and stored as DNA samples in the BRC Vatel collection (La Réunion). Each individual was amplified and genotyped using seven microsatellite markers developed by Gigant et al. (2012) (Table S2). Polymerase chain reaction (PCR) with fluorescently labeled primers was performed in a total volume of 25 µl using Promega kit (Promega, 2007) by mixing 12.5 µl of GoTaq Colorless Master Mix 2X, 2 μl of DNA solution (5 < DNA<250 ng), 0.4 μM of each primer, and 8.5 μl of nuclease‐free water. Amplifications were carried out under the following conditions: an initial denaturation step at 95°C for 2 min followed by 45 serial cycles of denaturation step at 95°C for 30 s, annealing at 57°C for 45 s, extension at 72°C for 1 min, and a final extension step at 72°C for 7 min. Genotyping of PCR fragments was performed by capillary electrophoresis with an automated sequencer 3130XL ABI Genetic Analyzer (Applied Biosystems). Allele sizes were scored using GENEIOUS v.7.0 (Olsen et al., 2014).

### 2.5 Genetic diversity

Genotypic diversity of each population was examined by dividing the number of unique genotypes G or “genets” by the total number of individuals N or “ramets” (Halkett et al., 2005). Clones were identified using the multilocus analysis of clonality in GenAlEx 6.5 (Peakall & Smouse, 2006). Clones were, then, excluded from the dataset for genetic analysis. The multilocus exact test *p*‐values based on Markov chain method (Guo & Thompson, 1992) in genepop v.4.7.5 (Raymond, 1995; Rousset, 2008) were used to test for deviation from Hardy–Weinberg equilibrium (HWE) in each population, using the default parameters (1,000 dememorizations, 100 batches, and 1,000 iterations per batch). GENEPOP was also used to perform probability tests for assessing the linkage disequilibrium (LD) between all pairs of loci in each population. The Holm's sequential Bonferroni procedure (Abdi, 2010) or SB correction, feasible under the “p.adjust” function in R software V3.6.1 (R Core Team, 2019), was performed to correct the levels of statistical significance (*p*‐value) for multiple hypothesis test of LD. The Waples method (Waples, 2015, 2018), examining the correlation between the mean squared correlation of allele frequencies at different gene locus (*r* ^2^) and the product of two fixation index (FST) for pairs of loci, was applied across all populations to test for a Wahlund or population structure effect on LD (Wahlund, 1928). GenAlEx 6.5 was used to calculate the total number of alleles for all loci in each population (Na), the mean observed number of alleles per locus (Al), and the number of private alleles per population (Pa). As the number of alleles in a sample is highly dependent on sample size, the mean allelic richness (Ar) as suggested by Mousadik and Petit (1996) was computed using FSTAT 2.9.4. (Goudet, 1995). We determined the observed heterozygosity (*H*_o_) and the mean expected heterozygosity under HWE (*H*_e_) over all loci using the R package GENEPOP v.4.7.5 (Raymond, 1995; Rousset, 2008). The correlations between Ar, He, and latitude were tested by performing Pearson correlation tests using R. The inbreeding coefficient FIS was calculated as FIS = 1−(*H*_o_/*H*_e_). Null allele frequencies at each locus across all populations were checked by INEst v.2.2 (Chybicki & Burczyk, 2009) using a Bayesian approach (IIM).

### 2.6 Genetic structure and differentiation

The “genets” dataset was used to estimate the global differentiation index (FST_g_) among populations (Weir & Cockerham, 1984) and the pairwise genetic differentiation (FST_p_) between pairs of populations using the GENEPOP package under R software. Analysis of molecular variance (AMOVA) (Excoffier et al., 1992) implemented in GenAlEx 6.5 (Peakall & Smouse, 2006) was conducted to assess the genetic differentiation level among and within populations and among populations from different habitat types. Each population was associated to its corresponding ecoregion of Madagascar based on geographic location.

Assignment of multilocus genotypes to different clusters was inferred by conducting Bayesian clustering analyses implemented in STRUCTURE (Pritchard et al., 2000) on the “genet” dataset, with ten independent runs for each value of K (1 to 10 for the complete dataset), a burn‐in period of 100,000 and 1,000,000 Markov chain Monte Carlo (MCMC) replications. To overcome sampling imbalance and thus correctly assign individuals to clusters, we used the model with admixture and independent allele frequencies and reduced the default alternative ancestry prior Alpha in STRUCTURE, as recommended by Wang (2017), with Alpha = 1/*K*. In our case, an Alpha equal to 1/10 was chosen, as the number of K tested was 10. An individual was considered to belong to a particular genetic group when at least 80% of its genome was assigned to that genetic group. The summary likelihood statistics Δ*K* (Evanno et al., 2005), which is the most used method to find the best K, was plotted. As the Δ*K* method alone is not recommended by some authors to determine the best clustering (Janes et al., 2017; Wang, 2017), the posterior probabilities of *K*, called “Ln *P*(*X*|*K*),” implemented in STRUCTURE were also examined (Pritchard et al., 2000). The best *K* usually corresponds to the highest value of mean Ln *P*(*X*|*K*) (Evanno et al., 2005; Pritchard et al., 2000; Rosenberg et al., 2001; Wang, 2017). Bar plot of genetic structuring obtained from STRUCTURE was generated using the POPHELPER package in R (Francis, 2017).

### 2.7 Floral differentiation

**FIGURE 2:**
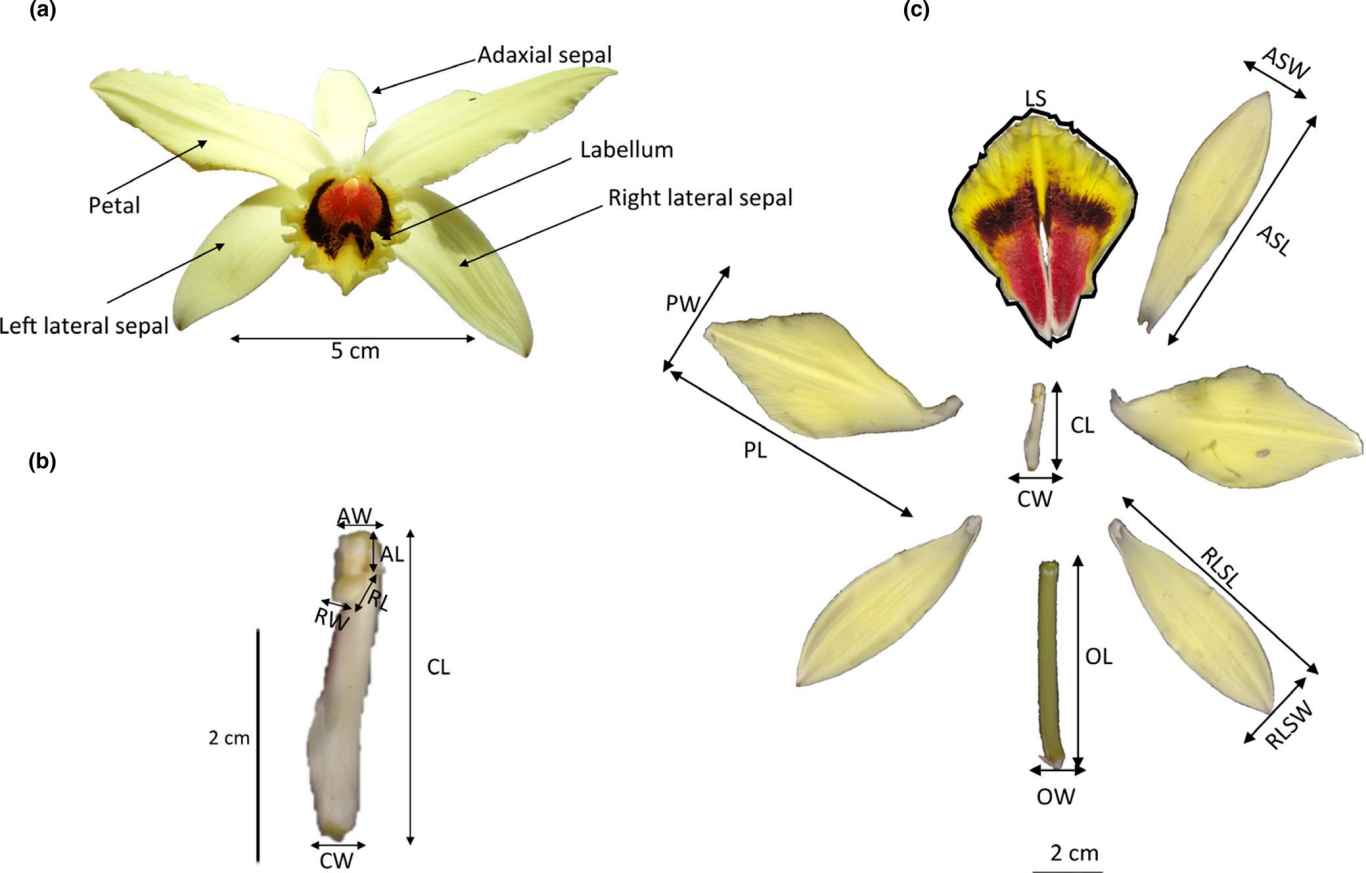
Floral measurements carried out on a *Vanilla* specimen with yellow flowers collected within the Montagne des Français (MDF, north) population. See text for morphological parameters abbreviations. Panel a: flower. Panel b: column. Panel c: flower parts

Eight populations assumed to represent all clusters from genetic structuring analysis were targeted during their flowering season based on bibliographical information (Andriamihaja et al., 2020): MSL and MND (October), BFD and MDF (November), and AJA, ATD, MLT, and AND (December) (Table S1). One herbarium specimen of each genetic group was deposited at the herbarium of the Botanical and Zoological Park of Tsimbazaza (N°51/20/MESupRES/SG/DGRS/PBZT/Flore). Twenty to thirty mature flowers per population were randomly sampled. In total, 198 flowers were dissected and classical taxonomic morphometric parameters were assessed (Gigant et al., 2014; Gigant, De Bruyn, et al., 2016; Petersson, 2015; Portères, 1954; Soto‐Arenas & Cribb, 2010). We directly measured in situ ten parameters using a digital caliper and a precision balance, that is, total flower weight (TFW), ovary weight (OWE), ovary length (OL), ovary width (OW), column length (CL), column width (CW), rostellum length (RL), rostellum width (RW), anther length (AL), and anther width (AW) (Figure 2).

Thereafter, flower parts were digitalized using a canoScan LiDE 120. The resulting images were processed under IMAGEJ software (Abràmoff et al., 2004) in order to measure complementary traits, that is, adaxial sepal width (ASW), adaxial sepal length (ASL), right lateral sepal length (RLSL), right lateral sepal width (RLSW), labellum surface (LS), petal length (PL), and petal width (PW) (Figure 2). Scanned flower images were also transformed into CIE (L*a*b*) color space according to the Kendal et al. (2013) script using the “convertColor” function in the grDevices package in R (R Core Team, 2019). The (L*) dimension values vary between 0 (black) and 100 (white reference) and represent the luminance. The (a*) and (b*) dimension values vary between −100 and 100 and represent the colors between red and green, and between yellow and blue, respectively (Najjar & Zagrouba, 2012). Color variables estimated were L* of inside of labellum (LIL), a* of inside of labellum (AIL), b* of inside of labellum (BIL), L* of outside face of sepal (LOFS), a* of outside face of sepal (AOFS), b* of outside face of sepal (BOFS), L* of inside face of petal (LIFP), a* of inside face of petal (AIFP), and b* of inside face of petal (BIFP). In total, 26 morphometric parameters were used in principal component analysis (PCA) using FACTOMINER (Lê et al., 2008) with R software. Three dimensions of PCA plot were generated with R package PCA3D (Weiner, 2019). PCA allows, by equal weighting of each character, reducing the number of variables in fewer dimensions and therefore investigating the overall morphological patterns. Means and standard deviations of the ten most explanatory variables from PCA were computed for each genetic group. Differences in these floral traits between groups were detected using a Bonferroni‐corrected pairwise *t* test. Multivariate analysis of variance (MANOVA) was also conducted to test the statistical significance of overall floral differences based on a priori groupings. All analyses were done with R software V3.5.1.

### 2.8 Geographic and environmental characterization of populations

Recorded GPS coordinates (Table S1) were used to calculate the geographic distances between *Vanilla* populations in Madagascar. Sample site elevation data were obtained from GPS (Table S1). To highlight the heterogeneity of habitats, 19 present‐day bioclimatic variables (means over thirty years) downloadable from the WorldClim website (https://www.worldclim.org/) were extracted for each sampling sites in Madagascar (Tables S3 and S4). In addition, the annual average values of maximum Normalized Difference Vegetation Index (NDVI) for each locality and four soil variables were also extracted from raster data at 250 m of resolution provided by GIMMS Global Agricultural Monitoring System (Becker‐Reshef et al., 2010) and World Reference Base for Soil Resources (WRB) (Tables S3 and S4) (Ribeiro et al., 2018). Pearson's correlation tests were performed to detect collinearity between variables, and a combination of variables with a correlation of less than 0.3 was chosen. Habitat heterogeneity between the 23 populations was shown by applying a PCA to these uncorrelated variables. Environmental dissimilarity between each pair of populations was obtained by calculating the Euclidian distances of the PC scores of the three first principal components (PCs).

As the landscape discontinuity could be an important factor to differentiation processes, we performed Monmonier algorithm analysis using BARRIER 2.2. (Manni et al., 2004) to identify where genetic barriers might occur. GPS coordinates of each population were entered to construct Voronoï tessellation that determines which populations are neighbors and adjacent. The pairwise genetic distance (FST_p_) was used to identify the top eleven potential barriers. Then, 100 resampled bootstrap Nei's standard genetic distances (Dn) matrices (Nei, 1972) were generated with MSA 4.05 (Dieringer & Schlötterer, 2003) for testing the robustness of each barrier (Manni et al., 2004).

### 2.9 Effect of environmental, morphological, and geographic distances on genetic divergence

To detect possible patterns of IBD and IBE, we performed multiple matrix regression with randomization (MMRR) as implemented in the R function “MMRR” (Wang, 2013) on the 23 populations. This approach is similar to Mantel test but can incorporate several distance matrices, thus providing an interpretable result in the form of a multiple regression equation (Wang, 2013). In the case of MMRR, significance values for the regression coefficient (*β*) and coefficient of determination (*R* ^2^) were estimated by performing 9,999 randomized permutation procedure (Wang, 2013). MMRR was also applied for testing the correlation between geographic and environmental distances. Genetic differentiation between populations was primarily estimated using pairwise FST values (Weir & Cockerham, 1984) using the “genet dataset” after discarding null alleles using FreeNa (Chapuis & Estoup, 2007). The geographic distance matrix was obtained with the SP package in R (Pebesma & Bivand, 2020, 2014). For environmental parameters, we used the Euclidean distance calculated from PC scores as explained in the previous section. Additionally, one powerful approach called “distance‐based redundancy analysis with randomization” (dbRDA), based on a linear ordination method that combines principal component analysis (PCA) and multiple linear regression (Legendre & Anderson, 1999), was applied using the “dbrda” function in the VEGAN package (Dixon, 2003) in R. Again, FST was tested against the geographic distances and population's PC scores from the PCA performed on the environmental data. Prior to IBD analysis, it was necessary to transform the geographic distances matrix into a continuous rectangular vector by principal coordinates analyses (PCoA) using the “pcnm” function included in the VEGAN package in R. Significance of the predictors was evaluated using multivariate F‐statistics with 9,999 permutations using the “ANOVA” function in VEGAN. Three different types of dbRDA were performed: (a) a global dbRDA using all explanatory variables (geographic and environmental distances), (b) marginal tests evaluating independently the relationship between the genetic distance matrix and each of the two explanatory variables (geographic and environmental factors), and (c) a partial dbRDA (conditional test) estimating the relative contribution of one explanatory variable (geographic distance or environmental factors) to genetic differentiation, while controlling the effect of the other one. The amount of variance explained by the intersection of IBD and IBE was obtained by variance partitioning using the function “varpart” included in VEGAN package. The adjusted coefficient of determination *R* ^2^_adj_ was calculated to evaluate the quality of the model. MMRR and dbRDA were also conducted on a reduced sampling of eight populations for which phenotypic data were available to test, in addition to IBD and IBE, a possible correlation between morphological distances and genetic distances (IBA). For that, a Pearson's correlation test on the 26 morphometric variables was carried out to choose a combination of uncorrelated variables (cor < 0.3). The PC scores of the first three principal components resulting from the PCA of these selected variables were, then, used to calculate the phenotypic distance between the eight populations in order to perform MMRR and dbRDA.

## 3 RESULTS

### 3.1 Genetic diversity

A total of 555 genets and 56 clones were identified among the 611 studied samples (ramets), representing approximately 9% of clonality. At the population level, the clonality rate was generally low (G/*N* > 0.7) except for AND population where genotypic diversity was only 0.27 (Table 1). The multilocus exact test p‐values for each population showed a significant deviation from HWE (*p*‐value < .05) (Table 1). For 22 populations, the observed heterozygosity (*H*_o_) was lower than the expected heterozygosity (*H*_e_), with positive FIS values, while Ho was higher than He (negative FIS value) for the AND population (Table 1). Overall diversity was high with He values ranging from 0.42 to 0.83. The seven primers revealed a high level of polymorphism with 262 alleles across the 23 studied populations. The total number of alleles over all loci per population varied from 16 (ANJ) to 112 (KRD), with a mean of 9 alleles/locus/population. Two populations (BBL, ZVB) had no private alleles, while the highest proportion was attributed to MND with 10 private alleles across all loci (Table 1). The mean allelic richness per locus (Ar) was lowest (2.86) in AND and highest (8.27) in KRD (Table 1). The Pearson's correlation showed that allelic richness (Ar) and expected heterozygosity (*H*_e_) increased slightly (rAr‐latitude = 0.43, rHe_latitude = 0.47) but significantly (*p* < .04) with latitude, with southern populations showing the lowest diversity and western populations showing the highest diversity (Table 1).

**TABLE 1:** Summary of genetic diversity at 7 microsatellite loci in 23 of leafless *Vanilla* populations from Madagascar

Pop	*N*	G/N	HWE	*H*_o_	*H*_e_	FIS	Na	Al	Pa	Ar
MDF	29	1.00	***	0.58	0.70	0.17	60	8.57 ± 1.65	5	5.57 ± 2.46
ANM	27	0.93	***	0.57	0.66	0.14	53	7.57 ± 1.46	1	5.52 ± 2.45
ANK	22	1.00	***	0.58	0.65	0.11	49	7.00 ± 1.11	2	5.04 ± 2.27
AMB	27	0.93	***	0.51	0.61	0.16	51	7.29 ± 1.34	2	4.95 ± 2.19
MND	41	0.93	***	0.52	0.67	0.22	84	12.00 ± 1.38	10	5.86 ± 2.17
BFD	24	1.00	***	0.62	0.80	0.22	73	10.43 ± 1.09	5	6.58 ± 1.39
ATS	27	0.93	***	0.58	0.82	0.30	80	11.43 ± 1.80	3	7.31 ± 2.65
MSL	41	0.93	***	0.61	0.82	0.26	95	13.57 ± 2.35	3	7.68 ± 2.86
CMK	26	1.00	***	0.57	0.79	0.28	78	11.14 ± 1.72	2	7.05 ± 2.55
BTM	34	1.00	***	0.63	0.74	0.15	75	10.71 ± 0.97	9	5.86 ± 1.28
BBL	26	1.00	***	0.59	0.82	0.28	92	13.14 ± 2.38	0	7.70 ± 3.10
AKF	24	1.00	***	0.68	0.81	0.16	96	13.71 ± 2.37	5	7.90 ± 3.05
NMK	24	0.96	***	0.68	0.78	0.13	93	13.29 ± 2.38	1	7.82 ± 3.17
KRD	33	1.00	***	0.67	0.83	0.20	112	16.00 ± 2.48	7	8.27 ± 2.70
KRM	29	0.90	***	0.41	0.53	0.22	51	7.29 ± 1.48	4	4.58 ± 2.52
AKL	25	0.80	***	0.61	0.81	0.25	57	8.14 ± 0.94	3	6.29 ± 1.57
ZVB	17	1.00	***	0.68	0.76	0.10	59	8.43 ± 1.31	0	6.39 ± 2.50
ATD	23	0.91	***	0.33	0.50	0.35	41	5.86 ± 1.18	3	4.07 ± 1.92
ANJ	24	0.83	**	0.23	0.35	0.32	16	2.29 ± 0.47	2	2.12 ± 1.02
MLT	38	0.82	***	0.45	0.61	0.27	55	7.86 ± 1.74	3	4.74 ± 2.53
SAG	9	1.00	*	0.29	0.43	0.34	25	3.57 ± 0.97	4	3.38 ± 2.35
MAN	11	0.82	***	0.49	0.62	0.21	33	4.71 ± 0.68	6	3.46 ± 1.58
AND	30	0.27	***	0.59	0.42	−0.40	20	2.86 ± 0.74	1	2.86 ± 1.95

Note

Pop, population; *N*, number of samples per population; G/N, genotypic diversity; HWE, results of test for departures from Hardy–Weinberg equilibrium (*p < .05, **p < .01, ***p < .001); *H*_o_, observed heterozygosity over all loci; *H*_e_, expected heterozygosity over all loci; FIS, fixation index; Na, total number of alleles for all loci; Al, mean number of alleles per locus ± *SD*; Pa, total number of private allele; and Ar, mean allelic richness per locus ± *SD* (Mousadik & Petit, 1996).

Of the 21 combinations of loci tested for LD across the populations, two‐locus combinations were significantly in LD after SB correction. LD analysis in each population revealed one‐locus to seven‐locus combinations in LD after SB correction in only 8 populations of the 23 studied, and with no pair of markers consistently in LD across all populations, as would be expected if LD resulted from physically linked loci (Slatkin, 2008). Therefore, all microsatellite loci were considered independent as shown by the original study of Gigant, De Bruyn, et al. (2016). A high frequency of null alleles ranging from 0.12 (HU03, HU07, HU08) to 0.26 (HU06) was detected.

### 3.2 Genetic structure, differentiation, and barriers

**FIGURE 3:**
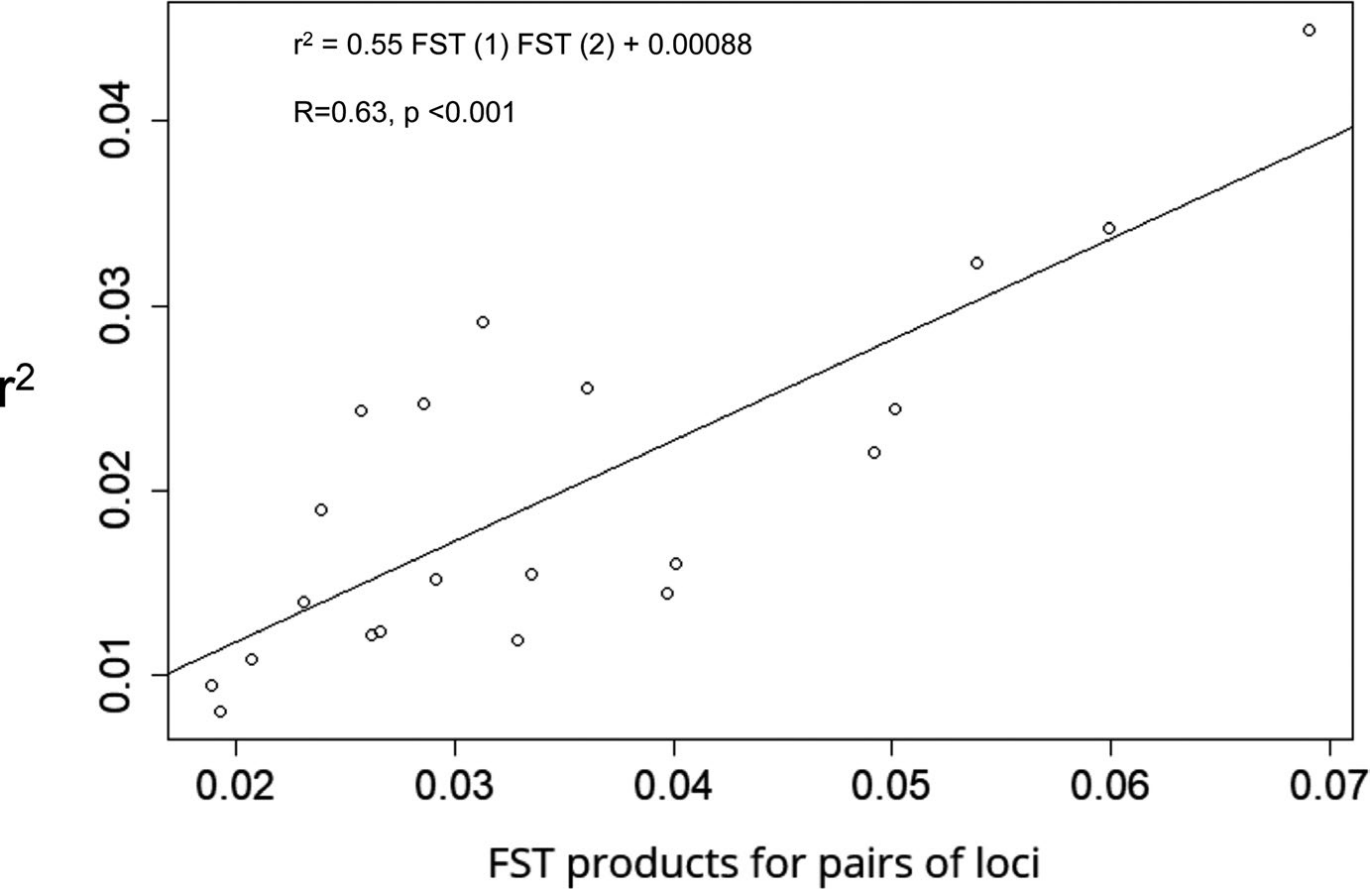
Scatterplot shows linear relationship between r2 (Waples, 2006) and the product of two FST values for 21 pairs of microsatellite loci across 23 populations of leafless *Vanilla* species from Madagascar

The global FST value calculated (0.19) indicated a significant (*p* < .001) overall differentiation among populations. Pairwise FST was all significant (*p* < .001), with high amplitude values ranging from 0.01 between two northwestern populations (BBL and MSL) up to 0.60 between AND (central highland) and ANJ (southwestern) (Table S5). A strong linear positive correlation (Pearson correlation coefficient = 0.81, r‐squared = 0.63; *p* < .001) was obtained between *r* ^2^ and the product of two FST values for pairs of loci, suggesting a substructure of the sample (Figure 3).

**FIGURE 4:**
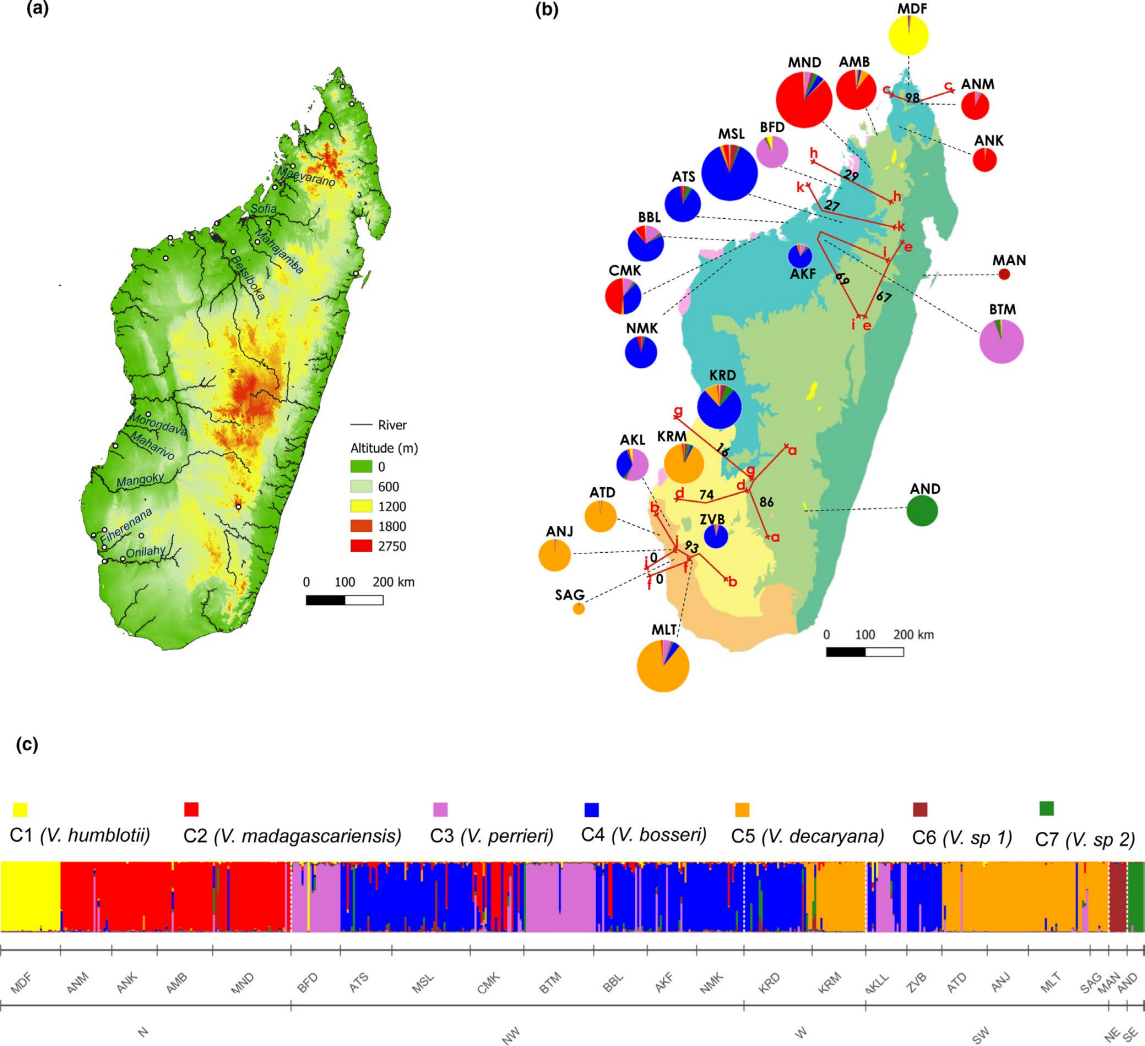
Genetic structuring and boundaries of leafless *Vanilla* populations from Madagascar. Panel a: topography of Madagascar with major rivers based on open street map data available on QGIS and USGS data (https://earthexplorer.usgs.gov/). The location of the 23 populations is indicated by circles. Panel b: distribution of leafless *Vanilla* genetic clusters in Madagascar based on Bayesian inference from STRUCTURE software and genetic barriers. Pie charts represent the mean proportion membership to *K* = 7 clusters and their sizes are proportional to sample sizes (*N*). Results of BARRIER analysis revealed regions of genetic discontinuity (in arrowed red lines) indicated by the letters a to k in descending order of FST values relative importance, with bootstrap values (in %) shown in black numbers calculated based on 100 Nei's standard genetic distances bootstrap matrices. Panel c: genetic assignment plot of individuals assessed by STRUCTURE program (*K* = 7). Each individual is represented by a vertical bar partitioned into K‐colored segments that represent the individual's probability of belonging to the cluster with that color. Corresponding species are indicated, according to population morphological analyses (Figure 5)

AMOVA estimated that 79% of the total genetic variation was found within populations, a moderate proportion (15%) was found among populations, and only 6% was found among ecoregions. The distribution of ∆*K* values in the Bayesian assignment of STRUCTURE showed a modal value at *K* = 4 and the maximum value of the posterior probability “Ln *P*(*X*|*K*)” corresponded to *K* = 7 (Figure S1). According to Wang (2017), with unbalanced sampling, it is preferable to choose the method of Pritchard et al. (2000) based on “Ln *P*(*X*|*K*)” to find the best *K*. Consequently, seven distinct genetic clusters were retained as the optimal *K* in STRUCTURE analysis (Figure 4b, c). If most populations were rather homogeneous in terms of genetic structure, some showed evidence of admixture, of which the most obvious were AKL (southwest) and CMK (northwest) (Figure 4b, c).

FST‐based analysis of genetic barriers revealed eleven boundaries. Six of them were well supported with bootstrap values exceeding 60%. These genetic barriers separated, in majority, the seven different genetic groups obtained from STRUCTURE (Figure 4b).

### 3.3 Floral morphology differentiation

**FIGURE 5:**
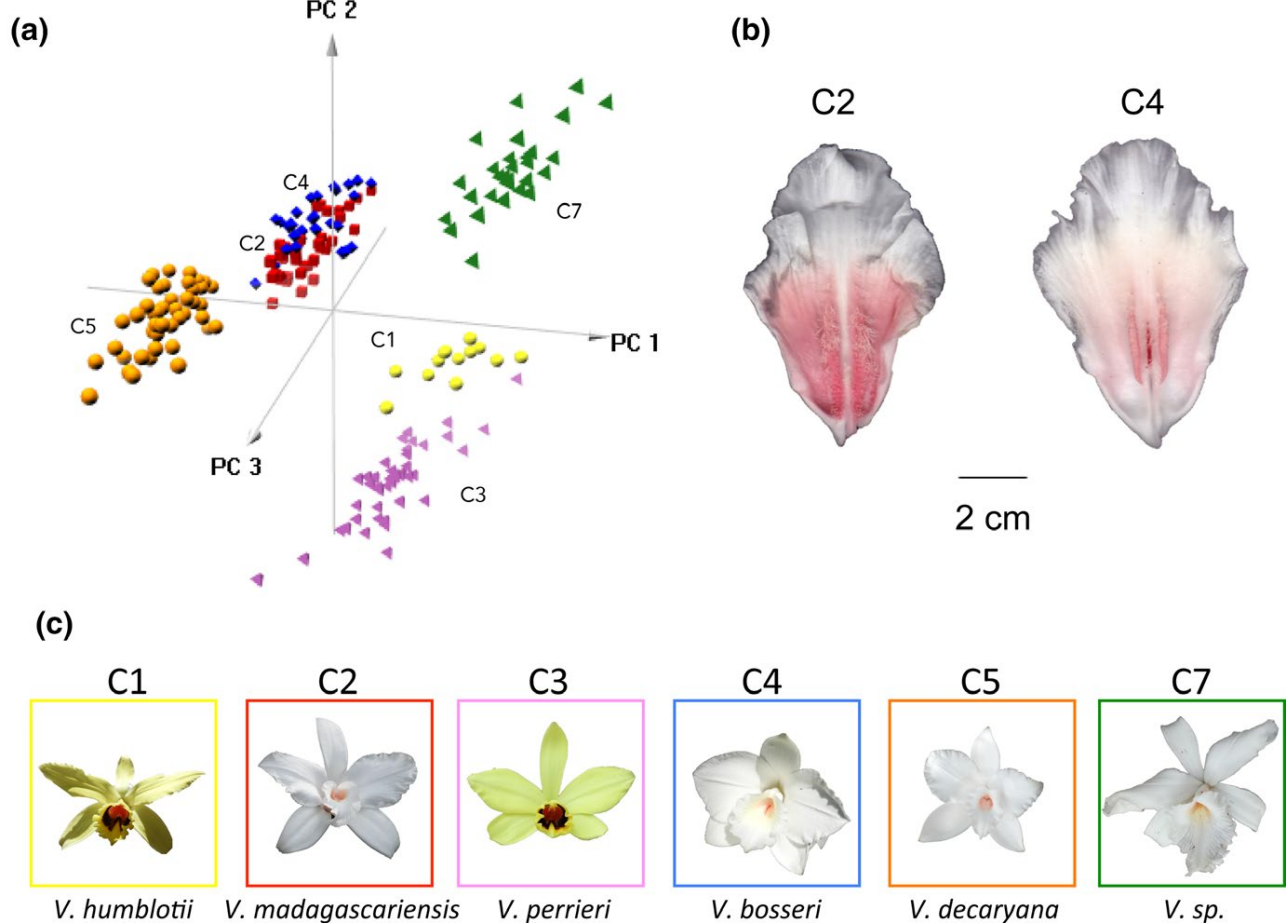
PCA of 26 quantitative floral morphometric characters and labellum hair qualitative character. (a) 3D plotting of the first three components of the PCA, with genetic groups from STRUCTURE analysis (*K* = 7) indicated by matching colors. C1: MDF; C2: MND; C3: BFD; C4: MSL; C5: ANJ, ATD, MLT; C7: AND. For the contribution of the 26 variables to the three axes of the PCA, see Figure S1. (b): Photograph of labellum of individuals from populations MND (C2) and MSL (C4) showing presence/absence of hairs. (c): Flower images representative of the 6 clusters: photographies by (C1) Johnson G. Andrianantenaina, (C2, C3, C4, C5): Cathucia F. Andriamihaja, (C7): Hoby Nomenjanahary

The first three principal components of PCA (Figure S2), explaining 80.6% of the total variation, were chosen to explain the total variation of 26 quantitative flowers traits, in relation with genetic clusters defined by STRUCTURE (*K* = 7). PCA1 explained 53.7% of the total variation and separated three clusters from three others by the weight and size of the flowers (Figure 5a, Figure S2). Color of the petals and of the inside face of labellum differentiated flowers from clusters 1 and 3 from the others on the second axis of the PCA with 19.3% of explained variance. Indeed, populations from these two clusters have yellow flowers, while the others have white flowers (Figure 5c). Color of the outside face of sepals and size of flowers contributed most strongly to PCA3 (7.6%) and separated populations from the south of Madagascar (cluster 5) and those situated in the north and west of Madagascar (clusters 2 and 4) (Figure 5a). Clusters 2 and 4 overlapped on the PCA axes. However, flowers from cluster 4 could readily be differentiated from those in cluster 2 by the absence of hairs inside the labellum, as detected during floral observation (Figure 5b). This morphological feature could not be included in the PCA analysis as it is a qualitative character. The MANOVA demonstrated significant differentiation of overall floral morphology between genetic groups (*p* < .0001). Means of the ten most explanatory variables of the first three PCA dimensions and results of pairwise *t* test are shown in Table 2. Seven to nine floral traits of the ten most discriminant variables varied significantly among all genetic groups, except for clusters 2 and 4, which presented many similarities as previously mentioned. The surface of the labellum was significantly different between all genetic groups.

**TABLE 2:** Comparison of ten floral parameters (mean ± SD) among six genetic groups derived from analysis with STRUCTURE. Means followed by the same letter in the same row are not significantly different (*p*‐value > .05)

Floral traits	C1	C2	C3	C4	C5	C7
Species	*VHUM*	*VMAD*	*VPER*	*VBOS*	*VDEC*	*VSP*
TFW (g)	6.08 ± 1.13^a^	2.88 ± 0.40^b^	4.22 ± 0.85^c^	3.33 ± 0.49^b^	1.76 ± 0.49^d^	7.93 ± 1.02 ^e^
BIFP	41.42 ± 5.86^a^	−2.61 ± 0.70^b^	58.48 ± 8.75^c^	−2.25 ± 0.78^b^	−2.39 ± 0.73^b^	−1.80 ± 0.64^b^
AIFP	−8.07 ± 1.06^a^	1.83 ± 0.35^b^	−10.31 ± 1.70^c^	2.25 ± 0.44^b^	1.76 ± 0.73^b^	1.80 ± 0.53^b^
LS	16.63 ± 2.28^a^	7.27 ± 0.83^b^	13.74 ± 1.94^c^	9.36 ± 1.23^d^	4.82 ± 0.36 ^e^	21.44 ± 2.50 ^f^
PL	7.46 ± 0.49^a^	5.84 ± 0.52^b^	6.55 ± 0.64^c^	5.88 ± 0.55^b^	4.23 ± 0.30^d^	8.79 ± 0.48 ^e^
RLSL	7.18 ± 0.36^a^	5.74 ± 0.51^b^	6.42 ± 0.60^c^	5.87 ± 0.54^b^	4.14 ± 0.20^d^	8.57 ± 0.40 ^e^
ASL	7.08 ± 0.57^a^	5.92 ± 0.59^b^	6.61 ± 0.70^a^	5.93 ± 0.51^b^	4.21 ± 0.30^c^	8.48 ± 0.52^d^
BIL	40.53 ± 1.97^a^	2.48 ± 0.88^b^	39.81 ± 6.64^a^	2.21 ± 1.32^b^	−0.72 ± 0.53^c^	0.82 ± 0.64^b,c^
OW	4.92 ± 0.52^a^	3.64 ± 0.26^b^	4.11 ± 0.46^c^	3.76 ± 0.29^b^	3.32 ± 0.30^d^	5.85 ± 0.58 ^e^
OWE	1.25 ± 0.43^a^	0.54 ± 0.11^b^	0.80 ± 0.23^c^	0.59 ± 0.10^b^	0.49 ± 0.12^b^	1.71 ± 0.29^d^

Note

BIFP: b* of inside face of petal, AIFP: a* of inside face of petal, BIL: b* of inside of labellum

Abbreviations: TFW, total floral weight; LS, labellum surface; PL, petal length; RSLS, right lateral sepal length; ASL, adaxial sepal length; OW, ovary width; OWE, ovary weight; *VHUM*, *V. humblotii*; *VMAD*, *V. madagascariensis*; *VPER*, *V. perrieri*; *VBOS*, *V. bosseri*; *VDEC*, *V. decaryana*; *and VSP*, *V. sp*.

### 3.4 Geographic, environmental, and morphological effects on genetic differentiation

**FIGURE 6:**
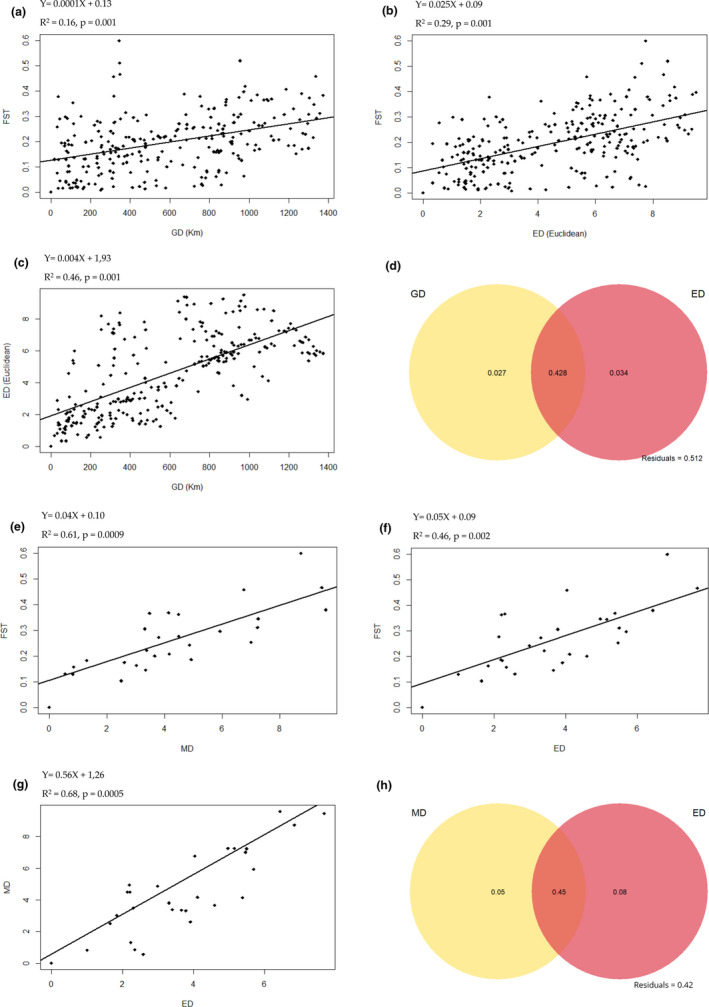
Multiple matrix regression with randomization (MMRR) analysis and distance‐based redundancy analysis (dbRDA) based on 23 populations (a, b, c, d) and 8 populations (e,f,g,h). (a) MMRR between genetic distance (FST) and geographic distance (GD), (b) MMRR between genetic distance (FST) and environmental distance (ED), (c) MMRR between environmental distance (ED) and geographic distance (GD). Regression lines are drawn for the three models a, b, c. (d) shows variance partitioning results of dbRDA on GD and ED. Geographic distance (GD) comprises eleven continuous rectangular vectors from principal coordinates analyses. Environmental factors include PC scores of three first components of the PCA (Figure S3). The overlapping zone represents the intersection between IBD and IBE. (e) MMRR between morphological distance (MD) and FST. (f) MMRR between ED and FST. (g). MMRR between MD and ED. Regression lines are drawn for the three models e,f,g. (h) shows variance partitioning results of dbRDA on MD and ED. Morphological factors include PC scores of the first three components of the PCA based on selected uncorrelated variables (Figure S4). Environmental factors include PC scores of three first components of the PCA (Figure S5). The overlapping zone represents the intersection between IBA and IBE

In general, PCA on the 14 uncorrelated environmental variables distributed samples between four types of habitat: east of Madagascar (MAN) with high NDVI and precipitation, north of Madagascar characterized by high annual temperature, west of Madagascar with high pH and temperature, and finally population from AND (central highlands of Madagascar) that can be distinguished from the others by its altitude (mean elevation: 887 m) (Figure S3). Scores of the three principal components (PCs) explaining 85.6% of the environmental variance were used to calculate environmental distances between the 23 populations. The results of MMRR on 23 populations revealed that genetic differentiation was weakly positively correlated to geographic distances (IBD) (*β* = 0.0001, *p* = .001) and with a higher coefficient of determination (*R* ^2^ = 0.29) to environmental distances (IBE) (*β* = 0.025, *p* = .001) (Figure 6a, b). However, geographic and environmental distances showed autocorrelation (Figure 6c). Marginal tests of dbRDA showed significant effect of IBD and IBE on genetic distance, whereas partial dbRDA revealed no pure contribution of IBD (*p* = .268) but a weak independent pattern of IBE (*R* ^2^_adj_ = 3.41%, *p* = .001) (Table 3). The intersection IBD ∩ IBE constituted the most important contributor to genetic divergence explaining 42.8% of the total variation (Figure 6d). Analyses carried out on the reduced sample (eight populations) indicated no contribution of geographic distance on genetic distance (no IBD) (Table 3) and eliminated its correlation with environmental distance. However, MMRR and dbRDA revealed a significant association between genetic distance and morphological distance (PC1) and between genetic distance and environmental distance (PC1) (Figure 6e,f) (Table 3). The PC1 axis from PCA of morphological data grouped together several variables related to flower size: TFW, PL, RLSL, ASL, OW, OWE, CL, and AL (Figure S4). The PC1 axis from PCA on environmental data assembles a pool of ecological variables related to temperature (BIO8, BIO10, BIO5), elevation, pH, and percentage of silt (Figure S5). However, environmental (PC1) and morphological predictors (PC1) were not significant after accounting for the influence of each other in partial dbRDA tests (Table 3), as they were highly intercorrelated (Figure 6g). The intersection IBE ∩ IBA constituted the most important contributor to genetic divergence explaining 45% of the total variation (Figure 6h).

**TABLE 3:** Results of distance‐based redundancy analyses (dbRDA) testing the effects of geographic distance (GD), environmental distance (ED), and morphological distance (MD) on genetic differentiation (FST) among 23 populations and 8 populations of leafless *Vanilla* species from Madagascar

dbRDA (23 populations)				dbRDA (8 populations)			
Marginal tests							
Variables	*F*	*p*	% of variables	Variables	*F*	*p*	% of variables
FST‐GD‐ED	2.91	0.007	48.83	FST‐GD	0.57	0.855	0
FST‐GD	2.66	0.006	45.42	FST‐ED (PC1)^c^	8.74	0.001	52.51
FST‐ED (PC1)^a^	3.94	0.013	11.80	FST‐MD (PC1)^d^	8.02	0.005	50.06
FST‐ED (PC2)^b^	12.33	0.001	33.99				

Partial dbRDA							
Variables/controlled	*F*	*p*	% of variables	Variables/controlled	*F*	*p*	% of variables
FST‐GD/ED	1.23	0.268	2.65	FST‐MD/ED	1.77	0.159	5.42
FST‐ED/GD	6.10	0.001	3.41	FST‐ED/MD	2.12	0.09	7.86

Note

In marginal tests of dbRDA, contribution of each explanatory variable (GD, ED, MD) to genetic differentiation was tested separately, while in partial (conditional) tests, the pure contribution of each factor was obtained after removing the covariate effects of the other factors. For the effects of ED and MD, only the PC axes that contributed significantly to genetic differentiation were represented. Explanatory variables: ^a^PC1 = BIO6, BIO4, BIO9, BIO16, BIO13, BIO7, BIO1, BIO3, BIO18; ^b^PC2 = BIO8, ELV, pH; ^c^PC1 = BIO8, BIO10, BIO5, ELV, pH, SILT; ^d^PC1 = TFW, PL, RLSL, ASL, OW, OWE, CL, AL.

## 4 DISCUSSION

### 4.1 Genetic diversity of leafless *Vanilla* populations

Numerous mechanisms can maintain genetic diversity, the most important of which are mutation, migration and balancing selection where population sizes play a major role (Frankham et al., 2010). Genetic diversity organization in plants is also highly dependent on the life cycle, reproduction system, pollination, and seed dispersion mechanisms. For example, long‐lived, allogamous taxa, with wind or ingested dispersed seeds, are more variable within populations than between, with high He (0.61–0.73) and lower FST (0.12–0.26) values, with an opposite organization for annual autogamous plants with gravity‐dispersed fruits (Nybom, 2004; Ronfort et al., 2005). AMOVA results suggested that genetic variation was mainly (79%) allocated within *Vanilla* populations in Madagascar. The levels of genetic diversity were relatively high for 20 populations out of the 23 (0.42 < He < 0.84, 3.45 < Ar < 7.65), with an overall low genetic differentiation (FST = 0.19). These results are compatible with what was expected for *Vanilla* species, which are long‐lived thanks to vegetative growth, mostly allogamous (Gigant et al., 2014; Gigant, De Bruyn, et al., 2016; Gigant, Rakotomanga, et al., 2016; Petersson, 2015), with most likely bee‐mediated pollination and suspected zoochorous seed dispersion (by bees or bats) (Gigant et al., 2011; Gigant, De Bruyn, et al., 2016). These results also agree with expectations for orchids species for which high He ranging from 0.3 up to 0.9 is reported (Chen et al., 2014; Soliva & Widmer, 2003; Stone et al., 2012; Swarts et al., 2009). Significant deviation from HW equilibrium was detected for all populations with a strong significant heterozygote deficit (FIS > 0) for 22 populations. This could be due to the high frequency of null alleles detected for all loci (0.12–0.26), although the substructure of some populations with different sympatric species (as will be further discussed), as well as possible inbreeding due to geitonogamy (as demonstrated for a *V. humblotii* Rchb. f. population in Mayotte, (Gigant, De Bruyn, et al., 2016), could be other factors explaining these deficits.

### 4.2 Genetic and morphological differentiation

At the scale of Madagascar, a strong significant population substructure was detected by the Wahlund effect test for overall individuals as suggested by Waples (2015, 2018) (Figure 3). The microsatellite‐based structuring corroborated this, by revealing seven well differentiated genetic groups (Figure 4b, 4c). Morphological analysis of Madagascar leafless *Vanilla* flowers revealed a similar grouping pattern to that found using microsatellite markers (STRUCTURE). Indeed, flower morphology was clearly specific for each of six STRUCTURE clusters (Figure 5), with size of flowers and particularly size of labellum as the determining feature of this differentiation (Table 2, Figure S2). In *Vanilla* plants, the difference in flower sizes is often a discriminating trait. For example, in the taxonomic review done by Soto‐Arenas and Cribb (2010), petal size was the determination key between *V. mexicana* and *V. ovata*, two closely related species from South America. Unfortunately, no flowers belonging to the genetic group C6 MAN could be observed. This morphometric pattern, combined to geographic distribution of known species (Figure 1b), allowed to clearly link five of the STRUCTURE clusters to the five botanical species already described in Madagascar (Allorge‐Boiteau, 2005, 2013; Cribb & Hermans, 2009; Portères, 1954). Flowers from clusters 1 and 3 matched the *V. humblotii* and *V. perrieri* Schltr. descriptions, respectively. Their weights and the amount of yellow in their flowers were significantly different according to our results (Figure 5, Table 2) and also as noted by Portères (1954). The presence of these two species in Madagascar has already been confirmed (Cribb & Hermans, 2009), although *V. humblotii* is supposedly endemic to the Comoros archipelago (Portères, 1954). The genetic groups with white flowers (C2, C4, C5, C7) could be differentiated mainly by the variation in their flower sizes (Table 2). Cluster 5 (ATD, MLT, KRD, SAG) flowers, which were the smallest (PL≈4 cm), were identified as morphologically close to the *V. decaryana* H. Perrier description by Allorge‐Boiteau (2005) in southern Madagascar. *V. bosseri* L. Allorge and *V. madagascariensis* Rolfe descriptions matched with clusters C4 and C2, respectively. Clusters 2 and 4, well differentiated on STRUCTURE analysis, are relatively close morphologically on the PCA, but they can be readily separated by the presence/absence of hairs on the labellum (Figure 5b). The absence of hairs is an originality of *V. bosseri* (C4) compared to all the other species (Allorge‐Boiteau, 2013; Petersson, 2015). Finally, the genetic group constituted by AND (cluster 7) was well differentiated and showed morphological traits different from the others that, however, have never been described in the literature.

Where species distribution overlap (Allorge‐Boiteau, 2013; Cribb & Hermans, 2009; Portères, 1954) (Figure 1), a combination of stratified populations and admixed individuals was detected, mainly in the northwest (CMK, ATS, BBL, MSL, AKF) and southwest (AKL, MLT) populations. These results, therefore, confirm possible sympatry and suggest that genetic exchanges are possible between sympatric species. Hybridization is a common phenomenon among *Vanilla* species (Bory et al., 2010; Divakaran et al., 2006; Gigant et al., 2011; Nielsen & Siegismund, 1999), with an absence of interspecific genetic incompatibility, particularly between closely related species (Bory et al., 2010).

### 4.3 Genetic discontinuities and speciation: forest and montane refugia, and riverine barriers

As genetic groups potentially corresponded to different species, diversification processes of leafless *Vanilla* populations in Madagascar could be interpreted as speciation mechanisms. Five mechanisms of speciation are recognized to be a pattern of diversification common for many taxa in Madagascar: ecological constraint influenced by bioclimatic disparities, western rainforest refugia, montane refugial diversification, riverine barriers, and watersheds (Vences et al., 2009). Interestingly, a genetic diversity center was revealed in the populations from west of Madagascar (highest He and Ar values, Table 1). Diversification could have resulted from a postglacial colonization from a western refuge area (Vences et al., 2009), to the north and the south, if considering the cline in diversity as the direction of spread (Hewitt, 1996). A similar scenario was suggested on the basis of paleoclimatic data for palm trees in the northeast of Madagascar (Rakotoarinivo et al., 2013), that have then recolonized the eastern corridor. The general pattern of genetic diversity showed a weak but significant latitudinal shift in the genetic diversity distribution (*r*_He‐Latitude_ = 0.47, *p* < .04) along the western coast of Madagascar. In addition to the probable effect of climatic oscillation explained previously, the lowest genetic diversity detected in southern populations might be partially linked to population size reductions (bottlenecks), as small and isolated populations are characterized by a low genetic diversity and linkage disequilibrium among loci (Frankham et al., 2010; Ouborg et al., 2006). Indeed, populations in the south of Madagascar are often exploited for medicinal use (Randrianarivony et al., 2017) and reduced by deforestation, as the two southwestern populations (ANJ and SAG) are found in unprotected areas close to villages. Low genetic diversity in fragmented habitats was also observed for some tropical tree populations in Africa (Andrianoelina et al., 2006, 2009; Farwig et al., 2007). Also, conjugated effects of deforestation and climate crisis, particularly emphasized in the south, could impact on seedlings recruitment (Moles & Westoby, 2004; Rasmussen, 2002).

Significant regions of genetic discontinuities were revealed with BARRIER analysis, between adjacent populations belonging to different genetic groups/species and indicating limited gene flow between these groups (Figure 4b). The most significant genetic barrier (0.23 < FST < 0.6) was observed between the genetic group C7 (AND), encountered in high altitude (up to 800 m) (Figure 4a, b) in a 49 ha conserved forest (Gould & Gabriel, 2015), and the others localized in low altitude, confirming probable differentiation by isolation. AND population could be the result of a “montane refugia speciation” supposing that population may have survived in high altitude during the climate shift, has adapted over time and remained isolated from the others (Fjeldsaå & Lovett, 1997; Vences et al., 2009). Moreover, the AND population showed a high clonality (73%) which probably explains the significant heterozygote excess (FIS < 0) detected (Halkett et al., 2005).

Rivers can constitute permanent geographic barriers that separated the continuous distribution range of populations leading to vicariant divergence (Vences et al., 2009). Five genetic boundaries identified by BARRIER analysis matched river separations. Indeed, population KRD and KRM belonging to different genetic groups are geographically separated by two small rivers in the west: Morondava and Maharivo (barrier g) (Figure 4a, b). The genetic barrier numbered d traces the path of the great Mangoky river separating the mid‐West population (KRM) from southwest populations (Figure 4a, b). Barriers h, i, and k can be linked to rivers in the north (Figure 4a, b). To what extent gene flow can be impeded by riverine barriers will have to be further assessed when better knowledge will be obtained regarding leafless *Vanilla* species pollinators and dispersers. Nevertheless, those riverine barriers might explain part of, but not all the genetic differentiation observed between populations/species. The occurrence of IBD, IBE, and IBA was therefore also assessed.

### 4.4 Combined effects of IBA and IBE as drivers of genetic divergence

Considering the geographic distribution of the genetic groups identified (Figure 4), some of them seem to be confined to well‐defined biomes (e.g., C5, C6 and C7) and AMOVA showed a weak but significant (6%) genetic variation between ecoregions. Tested separately on 23 populations, IBD and IBE played a significant role (*R* ^2^_adj_ = 45.42% and *R* ^2^_adj_ = 45.79%, respectively) on genetic differentiation. However, partial dbRDA showed a relatively weak (*R* ^2^_adj_ = 3.4%), but significant influence of environmental factors on genetic differentiation and no contribution of IBD. The combined effect of geographic and environmental distance (IBD ∩ IBE) was the most explanatory (*R* ^2^_adj_ = 42.8%). Indeed, spatial autocorrelation was detected between geographic and environmental distance (Figure 6c). This can be explained by the presence of clear ecoregions determined by the presence of a central mountain range and bioclimatic differences between west/east and north/south, which can lead to ecogeographic constraint speciation (Vences et al., 2009). The analysis of the reduced dataset (eight populations) revealed no IBD and no correlation between IBD and IBE. The suppression of this correlation allowed the pure contribution of these processes to be determined (Wang & Bradburd, 2014). Results from this reduced dataset confirmed the significant occurrence of IBE (*R* ^2^_adj_ = 52.51%) (with most discriminant variables being related to temperature, elevation, and soil pH) (Table 3). Moreover, significant IBA (*R* ^2^_adj_ = 50.06) (concerning flower size‐related measures) was also revealed in this dataset. But, there was a strong correlation between environment and morphological variables, making it difficult to disentangle their relative contribution to the genetic differentiation. Two evolutionary patterns can possibly explain this situation: Either IBA of floral traits could indicate pollinator‐mediated ecological selection, or ecological isolation could have led to drift‐induced morphological variations in flower size (Waser & Campbell, 2004). Pollinator‐mediated divergent ecological selection acts on flower traits (size, color, shape, odor…), therefore promoting isolation by adaptation (IBA) via floral isolation (Van der Niet et al., 2014). In orchids, pollinator specialization is common (Darwin, 1859; Peter & Johnson, 2020, 2014; Tremblay, 1992) and requires association between floral characteristics and insect traits (Micheneau et al., 2009; Petersson, 2015; Tremblay, 1992). For example, an association between spur length of some Angraecoid species and proboscis length of pollinators has been observed in Madagascar (Micheneau et al., 2009). Flower‐size variations in *Vanilla* species could be indicative of a pollinator specialization. Indeed, as noticed in Costa Rica, given the major size differences observed between *V. planifolia* and a newly described species *V. sotoarenasii*, it is unlikely that the same bee species can perform pollination efficiently in both species (Azofeifa‐Bolaños et al., 2017). Similarly, in Peru, small size bees (*Melipona* sp., *Euglossa* sp.) were shown to be unable to remove pollen from the large flowers of *V. grandiflora*, as opposed to *Eulaema meriana* (Householder et al., 2010; Lubinsky et al., 2006). Therefore, the eastern populations, with much larger flowers (as shown for AND), might be characterized by different pollinators than the western ones, and southwestern populations of *V. decaryana* (C5), with the smallest flowers, could be concerned as well by a certain level of pollinator specificity. However, various arguments are more in favor of the fact that most *Vanilla* species probably share the same pollinators in Madagascar. For *V. bosseri* in the Kirindy forest (KRD), Petersson (2015) identified three visiting bee species as possible pollinators: *Macrogalea ellioti*, *Liotrigona madecassa,* and *Liotrigona mahafalya*. These three bees have a north‐west‐south distribution (Pauly et al., 2001) matching the combined distributions of western *Vanilla* species. Another potential bee pollinator was also indicated based on floral morphology traits, *Lithurgus pullatus* which has a disjunct southwestern and northeastern distribution (Pauly et al., 2001). Moreover, in Mayotte, South Africa and Madagascar, leafless *Vanilla* species (*V. humblotii*, *V. roscheri*, *V. bosseri*, respectively) all have potential pollinator species from the Allodapini group (Gigant et al., 2014; Gigant, De Bruyn, et al., 2016; Petersson, 2015). In addition to bee species, the sunbird *Nectarinia coquerelli* was also identified as visitor and putative pollinator of *V. humblotii* in Mayotte (Gigant, De Bruyn, et al., 2016). At least one to two species belonging to the same genus, *Nectarinia notata* and *Nectarinia souimanga*, were observed in 31 dry forest sites in Madagascar (Raherilalao & Wilmé, 2008). High interannual variation in precipitation was observed in the west of Madagascar, which is associated with unpredictable patterns of flowering and fruiting (Dewar & Richard, 2007). This is consistent with the generalist hypothesis, at least for the western species: Some pollinators would compensate for the absence of the others to ensure species reproduction. Our results showing the existence of possible hybrids between some sympatric species in the west of Madagascar reinforces this hypothesis, as also demonstrated for two sympatric leafless *Vanilla* species in Puerto Rico (*V. barbellata* and *V. claviculata*) (Nielsen, 2000) for which the occurrence of hybrids suggested the same pollinator. In the genus *Vanilla*, in America, the known pollinators of wild species of the *V. planifolia* and *V. pompona* groups are similar, and they are all orchid‐bee species from the genera *Eugloss*a and *Eulaema* (Andriamihaja et al., 2020).

In plants, ecological isolation, apart from pollinator‐mediated divergent selection, is mainly based on the selection against migrants (Lowry et al., 2008; Nosil et al., 2005). Our results showed that discriminant environmental variables were related to temperature, altitude, and soil (pH). Altitude influence was previously discussed in the “montane refugia speciation” possibly involved for the high‐altitude population AND. Soil pH can affect mycorrhizal fungi composition (van Aarle et al., 2002; Porter et al., 1987). For orchids, establishment and growth is highly dependent on suitable mycorrhizal fungi for seed germination in addition to suitable pollinator populations for reproduction (Ackerman et al., 1996; Rasmussen, 2002; Rasmussen et al., 2015). In Puerto Rico, Cuba, and Costa Rica, different mycorrhizal fungi were detected in different sites associated with different *Vanilla* species, and fungal specificity for *Vanilla* seed germination and growth was shown (Porras‐Alfaro & Bayman, 2007). Characterizing the mycorrhizal fungi in each of the studied populations could allow to verify this, as done on orchids in the Central Highlands of Madagascar (Yokoya et al., 2015). Species differentiation may also be related to the different phenology of the species, influenced by climatic and ecological conditions such as temperature and altitude (Petrauski et al., 2019; Sobel et al., 2009). Indeed, *V. humblotii* and *V. perrieri* have overlapping phenologies (November‐April and November‐December, respectively) (Allorge‐Boiteau, 2005; Portères, 1954), but *V. bosseri* and *V. madagascariensis* flower earlier (September–October and June–October, respectively) (Allorge‐Boiteau, 2005, 2013; Portères, 1954). *V. decaryana* is reported to flower in December–January (Allorge‐Boiteau, 2013; Portères, 1954). Although the leafless *Vanilla* species *V. claviculata* and *V. barbellata* were shown to have overlapping phenologies in Puerto Rico, facilitating hybridization (Nielsen, 2000), differences in flowering periods with another sympatric species (*V. dilloniana*) have also been reported (Nielsen & Siegismund, 1999).

All results combined, the present study suggested a pattern of speciation possibly driven by the existence of riverine barriers and forest and montane refugees, in combination with isolation by ecology, as a result of adaptation to local habitats, possibly linked to local diversity in mycorrhizal fungi, and differences in flowering phenology, although pollinator‐mediated divergent selection in these *Vanilla* species could not be ruled out and might depend on the species concerned. Pollinator specificity could also be a consequence of floral differentiation consecutive to ecological speciation. Our results also showed the efficiency of a population genetics approach using highly variable microsatellite markers and the importance of large sample sizes for reliable estimates of the genetic relationship between recently diverged species, as shown for *Carappa* spp. (Duminil et al., 2012) and *Coffea* spp. (Razafinarivo et al., 2013).

This study is the first to provide an overview on the patterns of population genetic and morphological differentiation and to address issues related to the evolutionary mechanisms of several natural leafless *Vanilla* populations from Madagascar. It is nevertheless necessary to point out that this work has certain limitations, including the limited number of polymorphic microsatellite markers used, the small sample sizes of some populations (MAN, SAG, ZVB) and the absence of morphological data of one identified genetic group. The presence of new and significantly different genetic and morphological groups in the east, not yet botanically described, is interesting and will require further sampling and genetic studies in the eastern region, which is not specifically described as harboring leafless *Vanilla* species (Allorge‐Boiteau, 2005; Cribb & Hermans, 2009; Portères, 1954). Further studies on the Madagascar species reproduction biology (incompatibility, phenology, pollinators, seed dispersion, mycorrhizal fungi…) are needed. The Madagascar leafless *Vanilla* species will also need to be compared to sister species from the SWIO area to gain a broader phylogeographical view of their evolution.

## CONFLICT OF INTEREST

The authors declare no conflicts of interest.

## AUTHOR CONTRIBUTION

**Cathucia F. Andriamihaja:** Data curation (lead); Formal analysis (lead); Investigation (equal); Methodology (lead); Writing‐original draft (lead); Writing‐review & editing (equal). **Aro V. Ramarosandratana:** Conceptualization (equal); Data curation (equal); Funding acquisition (equal); Supervision (equal); Validation (equal); Writing‐review & editing (equal). **Michel Grisoni:** Conceptualization (equal); Funding acquisition (equal); Supervision (equal); Validation (equal); Writing‐review & editing (equal). **Vololoniaina H. Jeannoda:** Conceptualization (equal); Supervision (equal); Validation (supporting); Writing‐review & editing (equal). **Pascale Besse:** Conceptualization (lead); Data curation (equal); Formal analysis (equal); Funding acquisition (equal); Investigation (equal); Methodology (equal); Supervision (equal); Validation (equal); Writing‐original draft (equal); Writing‐review & editing (equal).

## Supporting information

Appendix S1

## Data Availability

Morphological measurement data and microsatellite data used to produce Figure 4, Figure 5, and Table 2 are accessible on UMR PVBMT Cirad dataverse (https://dataverse.cirad.fr/dataverse/pvbmt; https://dataverse.cirad.fr/dataset.xhtml?persistentId=doi:10.18167/DVN1/GWXWCB).

## References

[ece37224-bib-0001] Abdi, H. (2010). Holm’s Sequential Bonferroni Procedure. In N. Salkind (Ed.), Encyclopedia of research design (pp. 1–8). Sage.

[ece37224-bib-0002] Abràmoff, M. D. , Magalhães, P. J. , & Ram, S. J. (2004). Image processing with ImageJ. Biophotonics International, 11(7), 36–42.

[ece37224-bib-0003] Ackerman, J. D. , Sabat, A. , & Zimmerman, J. K. (1996). Seedling establishment in an epiphytic orchid: An experimental study of seed limitation. Oecologia, 106(2), 192–198. 10.1007/BF00328598 28307643

[ece37224-bib-0004] Allorge‐Boiteau, L. (2005). Les Vanilles Succulentes De Madagascar. Succulentes, 2, 3–11.

[ece37224-bib-0005] Allorge‐Boiteau, L. (2013). Une nouvelle espèce de vanille à Madagascar. Hommes Et Plantes, 85, 4–5.

[ece37224-bib-0006] Andriamihaja, C. F. , Ramarosandratana, A. V. , Grisoni, M. , Jeannoda, V. , & Besse, P. (2020). The Leafless *Vanilla* Species‐Complex from the South‐West Indian Ocean Region: A Taxonomic Puzzle and a Model for Orchid Evolution and Conservation Research. Diversity, 12(12), 443. 10.3390/d12120443

[ece37224-bib-0007] Andrianoelina, O. , Favreau, B. , Ramamonjisoa, L. , & Bouvet, J.‐M. (2009). Small effect of fragmentation on the genetic diversity of *Dalbergia monticola*, an endangered tree species of the eastern forest of Madagascar, detected by chloroplast and nuclear microsatellites. Annals of Botany, 104(6), 1231–1242. 10.1093/aob/mcp231 19773273 PMC2766213

[ece37224-bib-0008] Andrianoelina, O. , Rakotondraoelina, H. , Ramamonjisoa, L. , Maley, J. , Danthu, P. , & Bouvet, J.‐M. (2006). Genetic Diversity of *Dalbergia monticola* (Fabaceae) an Endangered Tree Species in the Fragmented Oriental Forest of Madagascar. Biodiversity & Conservation, 15(4), 1109–1128. 10.1007/s10531-004-2178-6

[ece37224-bib-0009] Arditti, J. , & Ghani, A. K. A. (2000). Tansley Review No. 110. New Phytologist, 145(3), 367–421. 10.1046/j.1469-8137.2000.00587.x 33862900

[ece37224-bib-0010] Arduino, P. , Verra, F. , Cianchi, R. , Rossi, W. , Corrias, B. , & Bullini, L. (1996). Genetic variation and natural hybridization between *Orchis laxiflora* and *Orchis palustris* (Orchidaceae). Plant Systematics and Evolution, 202(1), 87–109. 10.1007/BF00985819

[ece37224-bib-0011] Ayasse, M. , Stökl, J. , & Francke, W. (2011). Chemical ecology and pollinator‐driven speciation in sexually deceptive orchids. Phytochemistry, 72(13), 1667–1677. 10.1016/j.phytochem.2011.03.023 21497864

[ece37224-bib-0012] Azofeifa‐Bolaños, J. B. , Gigant, L. R. , Nicolás‐García, M. , Pignal, M. , Tavares‐González, F. B. , Hágsater, E. , Salazar‐Chávez, G. A. , Reyes‐López, D. , Archila‐Morales, F. L. , García‐García, J. A. , Da Silva, D. , Allibert, A. , Solano‐Campos, F. , Rodríguez‐Jimenes, G. D. C. , Paniagua‐Vásquez, A. , Besse, P. , Pérez‐Silva, A. , & Grisoni, M. (2017). A new *Vanilla* species from Costa Rica closely related to *V. planifolia* (Orchidaceae). European Journal of Taxonomy, 284, 1–26. 10.5852/ejt.2017.284

[ece37224-bib-0013] Barone Lumaga, M. R. , Pellegrino, G. , Bellusci, F. , Perrotta, E. , Perrotta, I. , & Musacchio, A. (2012). Comparative floral micromorphology in four sympatric species of *Serapias* (Orchidaceae). Botanical Journal of the Linnean Society, 169(4), 714–724. 10.1111/j.1095-8339.2012.01253.x

[ece37224-bib-0014] Batygina, T. B. , Bragina, E. A. , & Vasilyeva, V. E. (2003). The reproductive system and germination in orchids. Acta Biologica Cracoviensia Series Botanica, 45(2), 21–34.

[ece37224-bib-0015] Becker‐Reshef, I. , Justice, C. , Sullivan, M. , Vermote, E. , Tucker, C. , Anyamba, A. , Small, J. , Pak, E. D. , Masuoka, E. D. , Schmaltz, J. , Hansen, M. , Pittman, K. , Birkett, C. , Williams, D. , Reynolds, C. , & Doorn, B. (2010). Monitoring Global Croplands with Coarse Resolution Earth Observations: The Global Agriculture Monitoring (GLAM) Project. Remote Sensing, 2(6), 1589–1609. 10.3390/rs2061589

[ece37224-bib-0016] Bognounou, F. , Thiombiano, A. , Oden, P. C. , & Guinko, S. (2010). Seed provenance and latitudinal gradient effects on seed germination capacity and seedling establishment of five indigenous species in Burkina Faso. Tropical Ecology, 51(2), 207–220.

[ece37224-bib-0017] Bory, S. , Brown, S. , Duval, M.‐F. , & Besse, P. (2010). Evolutionary processes and diversification in the genus *Vanilla*. In E. Odoux , & M. Grisoni (Eds.), Vanilla (pp. 15–29). CRC Press.

[ece37224-bib-0018] Bosser, J. , & Lecoufle, M. (2011). Les orchidées de Madagascar. Biotope.

[ece37224-bib-0019] Bouetard, A. , Lefeuvre, P. , Gigant, R. L. , Bory, S. , Pignal, M. , Besse, P. , & Grisoni, M. (2010). Evidence of transoceanic dispersion of the genus *Vanilla* based on plastid DNA phylogenetic analysis. Molecular Phylogenetics and Evolution, 55(2), 621–630. 10.1016/j.ympev.2010.01.021 20109563

[ece37224-bib-0020] Burgess, N. D. , Hales, J. D. , Underwood, E. , Dinerstein, E. , Olson, D. , Itoua, I. , & Newman, K. (2004). Terrestrial Ecoregions of Africa and Madagascar: A Conservation Assessment. Island Press.

[ece37224-bib-0021] Byars, S. G. , Papst, W. , & Hoffmann, A. A. (2007). Local adaptation and cogradient selection in the alpine plant, *Poa Hiemata*, along a narrow altitudinal gradient. Evolution, 61(12), 2925–2941. 10.1111/j.1558-5646.2007.00248.x 17924954

[ece37224-bib-0022] Callmander, M. W. , Phillipson, P. B. , Schatz, G. E. , Andriambololonera, S. , Rabarimanarivo, M. , Rakotonirina, N. , Raharimampionona, J. , Chatelain, C. , Gautier, L. , & Lowry, P. P. (2011). The endemic and non‐endemic vascular flora of Madagascar updated. Plant Ecology and Evolution, 144(2), 121–125. 10.5091/plecevo.2011.513

[ece37224-bib-0023] Capparella, A. P. (1987). Effects of riverine barriers on genetic differentiation of Amazonian forest undergrowth birds (Peru). PhD Thesis. Louisiana State University. Retrieved from https://digitalcommons.lsu.edu/gradschool_disstheses/4346

[ece37224-bib-0024] Chapuis, M.‐P. , & Estoup, A. (2007). Microsatellite Null Alleles and Estimation of Population Differentiation. Molecular Biology and Evolution, 24(3), 621–631. 10.1093/molbev/msl191 17150975

[ece37224-bib-0025] Chen, Y.‐Y. , Bao, Z.‐X. , Qu, Y. , Li, W. , & Li, Z.‐Z. (2014). Genetic diversity and population structure of the medicinal orchid *Gastrodia elata* revealed by microsatellite analysis. Biochemical Systematics and Ecology, 54, 182–189. 10.1016/j.bse.2014.01.007

[ece37224-bib-0026] Chybicki, I. J. , & Burczyk, J. (2009). Simultaneous estimation of null alleles and inbreeding coefficients. Journal of Heredity, 100(1), 106–113. 10.1093/jhered/esn088 18936113

[ece37224-bib-0027] Cornet, A. (1973). Essai de cartographie bioclimatique à Madagascar. O.R.S.T.O.M.

[ece37224-bib-0028] Cribb, P. , & Hermans, J. (2007). The conservation of Madagascar’s orchids. A model for an integrated conservation project. Lankesteriana International Journal on Orchidology, 7(1–2), 255–261.

[ece37224-bib-0029] Cribb, P. , & Hermans, J. (2009). Field guide to the orchids of Madagascar. Royal Botanical Gardens.

[ece37224-bib-0030] Darwin, C. M. A. (1859). The origin of species by means of natural selection, or the preservation of favoured races in the struggle for life (2nd edn). John Murray. PMC5184128 30164232

[ece37224-bib-0031] De Frenne, P. , Graae, B. J. , Brunet, J. , Shevtsova, A. , De Schrijver, A. N. , Chabrerie, O. , Cousins, S. A. O. , Decocq, G. , Diekmann, M. , Hermy, M. , Heinken, T. , Kolb, A. , Nilsson, C. , Stanton, S. , & Verheyen, K. (2012). The response of forest plant regeneration to temperature variation along a latitudinal gradient. Annals of Botany, 109(5), 1037–1046. 10.1093/aob/mcs015 22345113 PMC3310497

[ece37224-bib-0032] Dewar, R. E. , & Richard, A. F. (2007). Evolution in the hypervariable environment of Madagascar. Proceedings of the National Academy of Sciences, 104(34), 13723–13727. 10.1073/pnas.0704346104 PMC1947998 17698810

[ece37224-bib-0033] Dieringer, D. , & Schlötterer, C. (2003). Microsatellite analyser (MSA): A platform independent analysis tool for large microsatellite data sets. Molecular Ecology Notes, 3(1), 167–169. 10.1046/j.1471-8286.2003.00351.x

[ece37224-bib-0034] Divakaran, M. , Babu, K. N. , Ravindran, P. N. , & Peter, K. V. (2006). Interspecific hybridization in *Vanilla* and molecular characterization of hybrids and selfed progenies using RAPD and AFLP markers. Scientia Horticulturae, 108(4), 414–422. 10.1016/j.scienta.2006.02.018

[ece37224-bib-0035] Dixon, P. (2003). VEGAN, a package of R functions for community ecology. Journal of Vegetation Science, 14(6), 927–930. 10.1111/j.1654-1103.2003.tb02228.x

[ece37224-bib-0036] Dressler, R. L. (1982). Biology of the Orchid Bees (*Euglossini*). Annual Review of Ecology and Systematics, 13(1), 373–394. 10.1146/annurev.es.13.110182.002105

[ece37224-bib-0037] Duminil, J. , Kenfack, D. , Viscosi, V. , Grumiau, L. , & Hardy, O. J. (2012). Testing species delimitation in sympatric species complexes: The case of an African tropical tree, *Carapa* spp. (Meliaceae). Molecular Phylogenetics and Evolution, 62(1), 275–285. 10.1016/j.ympev.2011.09.020 22019936

[ece37224-bib-0038] Evanno, G. , Regnaut, S. , & Goudet, J. (2005). Detecting the number of clusters of individuals using the software structure: A simulation study. Molecular Ecology, 14(8), 2611–2620. 10.1111/j.1365-294X.2005.02553.x 15969739

[ece37224-bib-0039] Excoffier, L. , Smouse, P. E. , & Quattro, J. M. (1992). Analysis of molecular variance inferred from metric distances among DNA haplotypes: Application to human mitochondrial DNA restriction data. Genetics, 131(2), 479–491. 10.1093/genetics/131.2.479 1644282 PMC1205020

[ece37224-bib-0040] Farwig, N. , Braun, C. , & Böhning‐Gaese, K. (2007). Human disturbance reduces genetic diversity of an endangered tropical tree, *Prunus africana* (Rosaceae). Conservation Genetics, 9(2), 317. 10.1007/s10592-007-9343-x

[ece37224-bib-0041] Feijó, A. , Wen, Z. , Cheng, J. , Ge, D. , Xia, L. , & Yang, Q. (2019). Divergent selection along elevational gradients promotes genetic and phenotypic disparities among small mammal populations. Ecology and Evolution, 9(12), 7080–7095. 10.1002/ece3.5273 31380035 PMC6662404

[ece37224-bib-0042] Fjeldsaå, J. , & Lovett, J. C. (1997). Geographical patterns of old and young species in African forest biota: The significance of specific montane areas as evolutionary centres. Biodiversity & Conservation, 6(3), 325–346. 10.1023/A:1018356506390

[ece37224-bib-0043] Francis, R. M. (2017). Pophelper: An R package and web app to analyse and visualize population structure. Molecular Ecology Resources, 17(1), 27–32. 10.1111/1755-0998.12509 26850166

[ece37224-bib-0044] Frankham, R. , Ballou, J. D. , & Briscoe, D. A. (2010). Introduction to conservation genetics. Cambridge University Press.

[ece37224-bib-0045] Gandon, S. , & Nuismer, S. L. (2009). Interactions between genetic drift, gene flow, and selection mosaics drive parasite local adaptation. The American Naturalist, 173(2), 212–224. 10.1086/593706 20374141

[ece37224-bib-0046] Garot, E. , Joët, T. , Combes, M.‐C. , & Lashermes, P. (2019). Genetic diversity and population divergences of an indigenous tree (*Coffea mauritiana*) in Reunion Island: Role of climatic and geographical factors. Heredity, 122(6), 833–847. 10.1038/s41437-018-0168-9 30478354 PMC6781115

[ece37224-bib-0047] Ghalambor, C. K. , McKay, J. K. , Carroll, S. P. , & Reznick, D. N. (2007). Adaptive versus non‐adaptive phenotypic plasticity and the potential for contemporary adaptation in new environments. Functional Ecology, 21(3), 394–407. 10.1111/j.1365-2435.2007.01283.x

[ece37224-bib-0048] Gigant, R. L. , Bory, S. , Grisoni, M. , & Besse, P. (2011). Biodiversity and evolution in the *Vanilla* genus. In The Dynamical Processes of Biodiversity: Case Studies of Evolution and Spatial Distribution (pp. 1–26). InTech.

[ece37224-bib-0049] Gigant, R. , Brugel, A. , De Bruyn, A. , Risterucci, A. M. , Guiot, V. , Viscardi, G. , Humeau, L. , Grisoni, M. , & Besse, P. (2012). Nineteen polymorphic microsatellite markers from two African *Vanilla* species: Across‐species transferability and diversity in a wild population of *V. humblotii* from Mayotte. Conservation Genetics Resources, 4(1), 121–125. 10.1007/s12686-011-9489-1

[ece37224-bib-0050] Gigant, R. L. , De Bruyn, A. , Church, B. , Humeau, L. , Gauvin‐Bialecki, A. , Pailler, T. , Grisoni, M. , & Besse, P. (2014). Active sexual reproduction but no sign of genetic diversity in range‐edge populations of *Vanilla roscheri* Rchb. f. (Orchidaceae) in South Africa. Conservation Genetics, 15(6), 1403–1415. 10.1007/s10592-014-0626-8

[ece37224-bib-0051] Gigant, R. L. , De Bruyn, A. , M'sa, T. , Viscardi, G. , Gigord, L. , Gauvin‐Bialecki, A. , Pailler, T. , Humeau, L. , Grisoni, M. , & Besse, P. (2016). Combining pollination ecology and fine‐scale spatial genetic structure analysis to unravel the reproductive strategy of an insular threatened orchid. South African Journal of Botany, 105, 25–35. 10.1016/j.sajb.2016.02.205

[ece37224-bib-0052] Gigant, R. L. , Rakotomanga, N. , Citadelle, G. , Silvestre, D. , Grisoni, M. , & Besse, P. (2016). Microsatellite markers confirm self‐pollination and autogamy in wild populations of Vanilla mexicana Mill. (syn. V. inodora) (Orchidaceae) in the island of Guadeloupe. In I. Abdurakhmonov (Ed.), Microsatellite Markers (pp. 73–93). InTechOpen.

[ece37224-bib-0053] Givnish, T. J. , Spalink, D. , Ames, M. , Lyon, S. P. , Hunter, S. J. , Zuluaga, A. , Iles, W. J. D. , Clements, M. A. , Arroyo, M. T. K. , Leebens‐Mack, J. , Endara, L. , Kriebel, R. , Neubig, K. M. , Whitten, W. M. , Williams, N. H. , & Cameron, K. M. (2015). Orchid phylogenomics and multiple drivers of their extraordinary diversification. Proceedings of the Royal Society B: Biological Sciences, 282(1814), 20151553. 10.1098/rspb.2015.1553 PMC4571710 26311671

[ece37224-bib-0054] Gomez, C. , Dussert, S. , Hamon, P. , Hamon, S. , de Kochko, A. , & Poncet, V. (2009). Current genetic differentiation of *Coffea canephora* Pierre ex A. Froehn in the Guineo‐Congolian African zone: Cumulative impact of ancient climatic changes and recent human activities. BMC Evolutionary Biology, 9(1), 167. 10.1186/1471-2148-9-167 19607674 PMC2717059

[ece37224-bib-0055] Goodman, S. M. , & Benstead, J. P. (2005). Updated estimates of biotic diversity and endemism for Madagascar. Oryx, 39(1), 73–77. 10.1017/S0030605305000128

[ece37224-bib-0056] Goudet, J. (1995). FSTAT (Version 1.2): A Computer Program to Calculate F‐Statistics. Journal of Heredity, 86(6), 485–486. 10.1093/oxfordjournals.jhered.a111627

[ece37224-bib-0057] Gould, L. , & Gabriel, D. N. (2015). Wet and dry season diets of the Endangered *Lemur catta* (ring‐tailed lemur) in two mountainous rocky outcrop forest fragments in south‐central Madagascar. African Journal of Ecology, 53(3), 320–330. 10.1111/aje.12186

[ece37224-bib-0058] Guo, S. W. , & Thompson, E. A. (1992). Performing the Exact Test of Hardy‐Weinberg Proportion for Multiple Alleles. Biometrics, 48(2), 361–372. 10.2307/2532296 1637966

[ece37224-bib-0059] Halkett, F. , Simon, J.‐C. , & Balloux, F. (2005). Tackling the population genetics of clonal and partially clonal organisms. Trends in Ecology & Evolution, 20(4), 194–201. 10.1016/j.tree.2005.01.001 16701368

[ece37224-bib-0060] Hewitt, G. M. (1996). Some genetic consequences of ice ages, and their role in divergence and speciation. Biological Journal of the Linnean Society, 58(3), 247–276. 10.1111/j.1095-8312.1996.tb01434.x

[ece37224-bib-0061] Householder, E. , Janovec, J. , Mozambite, A. B. , Maceda, J. H. , Wells, J. , & Valega, R. (2010). Diversity, natural history, and conservation of *Vanilla* (orchidaceae) in Amazonian wetlands of Madre De Dios, Peru. Journal of Botanical Rsearch Institute of Texas, 4(1), 227–243.

[ece37224-bib-0062] Janes, J. K. , Miller, J. M. , Dupuis, J. R. , Malenfant, R. M. , Gorrell, J. C. , Cullingham, C. I. , & Andrew, R. L. (2017). The *K* = 2 conundrum. Molecular Ecology, 26(14), 3594–3602. 10.1111/mec.14187 28544181

[ece37224-bib-0063] Jaros, U. , Fischer, G. A. , Pailler, T. , & Comes, H. P. (2016). Spatial patterns of AFLP diversity in *Bulbophyllum occultum* (Orchidaceae) indicate long‐term refugial isolation in Madagascar and long‐distance colonization effects in La Réunion. Heredity, 116(5), 434–446. 10.1038/hdy.2016.1 26883184 PMC4834385

[ece37224-bib-0064] Johnson, M. T. J. , Prashad, C. M. , Lavoignat, M. , & Saini, H. S. (2018). Contrasting the effects of natural selection, genetic drift and gene flow on urban evolution in white clover (*Trifolium repens*). Proceedings of the Royal Society B: Biological Sciences, 285(1883), 20181019. 10.1098/rspb.2018.1019 PMC6083247 30051843

[ece37224-bib-0065] Kendal, D. , Hauser, C. E. , Garrard, G. E. , Jellinek, S. , Giljohann, K. M. , & Moore, J. L. (2013). Quantifying Plant Colour and Colour Difference as Perceived by Humans Using Digital Images. PLoS One, 8(8), e72296. 10.1371/journal.pone.0072296 23977275 PMC3748102

[ece37224-bib-0066] Lê, S. , Josse, J. , & Husson, F. (2008). FactoMineR : An *R* package for multivariate analysis. Journal of Statistical Software, 25(1), 1–18. 10.18637/jss.v025.i01

[ece37224-bib-0067] Legendre, P. , & Anderson, M. J. (1999). Distance‐based redundancy analysis: Testing multispecies responses in multifactorial ecological experiments. Ecological Monographs, 69(1), 1–24. 10.1890/0012-9615(1999)069[0001:DBRATM]2.0.CO;2

[ece37224-bib-0068] Lekberg, Y. , Roskilly, B. , Hendrick, M. F. , Zabinski, C. A. , Barr, C. M. , & Fishman, L. (2012). Phenotypic and genetic differentiation among yellow monkeyflower populations from thermal and non‐thermal soils in Yellowstone National Park. Oecologia, 170(1), 111–122. 10.1007/s00442-012-2297-9 22437908

[ece37224-bib-0069] Liu, W. , Zhao, Y. , Qi, D. , You, J. , Zhou, Y. , & Song, Z. (2018). The Tanggula Mountains enhance population divergence in *Carex moorcroftii* : A dominant sedge on the Qinghai‐Tibetan Plateau. Scientific Reports, 8(1), 2741. 10.1038/s41598-018-21129-y 29426823 PMC5807306

[ece37224-bib-0070] Lowry, D. B. , Modliszewski, J. L. , Wright, K. M. , Wu, C. A. , & Willis, J. H. (2008). The strength and genetic basis of reproductive isolating barriers in flowering plants. Philosophical Transactions of the Royal Society B: Biological Sciences, 363(1506), 3009–3021. 10.1098/rstb.2008.0064 PMC2607309 18579478

[ece37224-bib-0071] Lowry, D. B. , Rockwood, R. C. , & Willis, J. H. (2008). Ecological reproductive isolation of coast and inland races of *Mimulus Guttatus* . Evolution, 62(9), 2196–2214. 10.1111/j.1558-5646.2008.00457.x 18637837 PMC11110535

[ece37224-bib-0072] Lubinsky, P. , Van Dam, M. , & Van Dam, A. (2006). Pollination of *Vanilla* and evolution in the Orchidaceae. Lindleyana, 75, 926–929.

[ece37224-bib-0073] Mallet, B. , Martos, F. , Blambert, L. , Pailler, T. , & Humeau, L. (2014). Evidence for Isolation‐by‐Habitat among Populations of an Epiphytic Orchid Species on a Small Oceanic Island. PLoS One, 9(2), e87469. 10.1371/journal.pone.0087469 24498329 PMC3911949

[ece37224-bib-0074] Manni, F. , Guérard, E. , & Heyer, E. (2004). Geographic Patterns of (Genetic, Morphologic, Linguistic) Variation: How Barriers Can Be Detected by Using Monmonier's Algorithm. Human Biology, 76(2), 173–190. 10.1353/hub.2004.0034 15359530

[ece37224-bib-0075] Médail, F. , & Diadema, K. (2009). Glacial refugia influence plant diversity patterns in the Mediterranean Basin. Journal of Biogeography, 36(7), 1333–1345. 10.1111/j.1365-2699.2008.02051.x

[ece37224-bib-0076] Micheneau, C. , Johnson, S. D. , & Fay, M. F. (2009). Orchid pollination: From Darwin to the present day. Botanical Journal of the Linnean Society, 161(1), 1–19. 10.1111/j.1095-8339.2009.00995.x

[ece37224-bib-0077] Moles, A. T. , & Westoby, M. (2004). Seed mass and seedling establishment after fire in Ku‐ring‐gai Chase National Park, Sydney, Australia. Austral Ecology, 29(4), 383–390. 10.1111/j.1442-9993.2004.01374.x

[ece37224-bib-0078] Mousadik, A. E. , & Petit, R. J. (1996). High level of genetic differentiation for allelic richness among populations of the argan tree [*Argania spinosa* (L.) Skeels] endemic to Morocco. Theoretical and Applied Genetics., 92, 832–839. 10.1007/BF00221895 24166548

[ece37224-bib-0079] Najjar, A. , & Zagrouba, E. (2012). Flower image segmentation based on color analysis and a supervised evaluation. International Conference on Communications and Information Technology (ICCIT), 2012, 397–401. 10.1109/ICCITechnol.2012.6285834

[ece37224-bib-0080] Nei, M. (1972). Genetic distance between populations. The American Naturalist, 106(949), 283–292. 10.1086/282771

[ece37224-bib-0081] Nielsen, L. R. (2000). Natural hybridization between *Vanilla claviculata* (W.Wright) Sw. and *V. barbellata* Rchb.f. (Orchidaceae): Genetic, morphological, and pollination experimental data. Botanical Journal of the Linnean Society, 133(3), 285–302. 10.1111/j.1095-8339.2000.tb01547.x

[ece37224-bib-0082] Nielsen, L. R. , & Siegismund, H. R. (1999). Interspecific differentiation and hybridization in *Vanilla* species (Orchidaceae). Heredity, 83(5), 560–567. 10.1038/sj.hdy.6885880 10620028

[ece37224-bib-0083] Noguerales, V. , Cordero, P. J. , & Ortego, J. (2016). Hierarchical genetic structure shaped by topography in a narrow‐endemic montane grasshopper. BMC Evolutionary Biology, 16(1), 96. 10.1186/s12862-016-0663-7 27149952 PMC4858822

[ece37224-bib-0084] Nosil, P. , Egan, S. P. , & Funk, D. J. (2008). Heterogeneous genomic differentiation between walking‐stick ecotypes: “isolation by adaptation” and multiple roles for divergent selection. Evolution, 62(2), 316–336. 10.1111/j.1558-5646.2007.00299.x 17999721

[ece37224-bib-0085] Nosil, P. , Vines, T. H. , & Funk, D. J. (2005). Reproductive isolation caused by natural selection against immigrants from divergent habitats. Evolution, 59(4), 705–719. 10.1111/j.0014-3820.2005.tb01747.x 15926683

[ece37224-bib-0086] Nybom, H. (2004). Comparison of different nuclear DNA markers for estimating intraspecific genetic diversity in plants. Molecular Ecology, 13(5), 1143–1155. 10.1111/j.1365-294X.2004.02141.x 15078452

[ece37224-bib-0087] Olsen, C. , Qaadri, K. , & Place, P. (2014). Geneious R7: A bioinformatics platform for biologists. In Poster presentation in the Plant and Animal Genome XXII Conference presented at the San Diego, CA, USA. https://www.eposters.net/pdfs/geneious‐r7‐a‐bioinformatics‐platform‐for‐biologists.pdf

[ece37224-bib-0088] Olsson, K. , & Ågren, J. (2002). Latitudinal population differentiation in phenology, life history and flower morphology in the perennial herb *Lythrum salicaria* . Journal of Evolutionary Biology, 15(6), 983–996. 10.1046/j.1420-9101.2002.00457.x

[ece37224-bib-0089] Orsini, L. , Vanoverbeke, J. , Swillen, I. , Mergeay, J. , & Meester, L. D. (2013). Drivers of population genetic differentiation in the wild: Isolation by dispersal limitation, isolation by adaptation and isolation by colonization. Molecular Ecology, 22(24), 5983–5999. 10.1111/mec.12561 24128305

[ece37224-bib-0090] Otero, J. T. , Flanagan, N. S. , Herre, E. A. , Ackerman, J. D. , & Bayman, P. (2007). Widespread mycorrhizal specificity correlates to mycorrhizal function in the neotropical, epiphytic orchid *Lonopsis utricularioides* (Orchidaceae). American Journal of Botany, 94(12), 1944–1950. 10.3732/ajb.94.12.1944 21636389

[ece37224-bib-0091] Ouborg, N. J. , Vergeer, P. , & Mix, C. (2006). The rough edges of the conservation genetics paradigm for plants. Journal of Ecology, 94(6), 1233–1248. 10.1111/j.1365-2745.2006.01167.x

[ece37224-bib-0092] Pansarin, E. R. , & Ferreira, A. W. C. (2019). Elucidating cryptic sympatric speciation in terrestrial orchids. BioRxiv, 828491. 10.1101/828491

[ece37224-bib-0093] Pauly, A. , Brooks, R. W. , Nilsson, A. , Pesenko, Y. A. , Eardley, C. D. , Terzo, M. , & Munzinger, J. (2001). Hymenoptera Apoidea de Madagascar et des îles voisines. Musée royal de l’Afrique centrale. Retrieved from https://www.pemberleybooks.com/product/hymenoptera‐apoidea‐de‐madagascar‐et‐des‐les‐voisines/18946/

[ece37224-bib-0094] Peakall, R. , & Smouse, P. E. (2006). GenAlEx 6.5: Genetic analysis in Excel. Population genetic software for teaching and research–an update. Molecular Ecology Notes, 6(1), 288–295. 10.1111/j.1471-8286.2005.01155.x PMC3463245 22820204

[ece37224-bib-0095] Pebesma and Bivand, 2020 Pebesma, E. , & Bivand, R. (2020). Package ‘sp.’ Retrieved from The Comprehensive R Archive Network. https://cran.r‐project.org/web/packages/sp/sp.pdf

[ece37224-bib-0096] Peter, C. I. , & Johnson, S. D. (2014). A pollinator shift explains floral divergence in an orchid species complex in South Africa. Annals of Botany, 113(2), 277–288. 10.1093/aob/mct216 24107684 PMC3890387

[ece37224-bib-0097] Petersson, L. (2015). Pollination biology of the endemic orchid Vanilla bosseri in Madagascar. Master Thesis. Uppsala University, Disciplinary Domain of Science and Technology, Biology Education Center. Retrieved from http://urn.kb.se/resolve?urn=urn:nbn:se:uu:diva‐265232

[ece37224-bib-0098] Petrauski, L. , Owen, S. F. , Constantz, G. D. , & Anderson, J. T. (2019). Changes in flowering phenology of *Cardamine concatenata* and *Erythronium americanum* over 111 years in the Central Appalachians. Plant Ecology, 220(9), 817–828. 10.1007/s11258-019-00956-7

[ece37224-bib-0099] Phillipson, P. B. , Schatz, G. E. , & Li, P. P. L. (2006) A catalogue of the vascular plants of Madagascar. In S. A. Ghazanfar , & H. J. Beentje (Eds.), Proceedings of the 17th AETFAT Congress (pp. 613–627). Royal Botanic Gardens Kew.

[ece37224-bib-0100] Porras‐Alfaro, A. , & Bayman, P. (2007). Mycorrhizal fungi of *Vanilla*: Diversity, specificity and effects on seed germination and plant growth. Mycologia, 99(4), 510–525. 10.1080/15572536.2007.11832545 18065002

[ece37224-bib-0101] Porter, W. M. , Robson, A. D. , & Abbott, L. K. (1987). Field survey of the distribution of vesicular‐arbuscular mycorrhizal fungi in relation to soil pH. The Journal of Applied Ecology, 24(2), 659. 10.2307/2403900

[ece37224-bib-0102] Portères, R. (1954). Le Genre *Vanilla* et ses Espèces. In G. Bouriquet (Ed.), Le vanillier et la vanille dans le monde (pp. 94–920). Editions P. Lechevalier.

[ece37224-bib-0103] Pritchard, J. K. , Stephens, M. , & Donnelly, P. (2000). Inference of Population Structure Using Multilocus Genotype Data. Genetics, 155(2), 945–959. 10835412 10.1093/genetics/155.2.945 PMC1461096

[ece37224-bib-0104] Promega (2007). Usage information of Gotaq Colorless Master Mix. Retrieved from Resources website: https://www.promega.com/‐/media/files/resources/protocols/product‐information‐sheets/g/gotaq‐colorless‐master‐mix‐m713.pdf?la=en

[ece37224-bib-0105] R Core Team (2019). R: A language and environment for statistical computing (Version 3.6.1). R Foundation for Statistical Computing.

[ece37224-bib-0106] Raherilalao, M. J. , & Wilmé, L. (2008). L’avifaune des forêts sèches malgaches. In S. M. Goodman , & L. Wilmé (Eds.), Dans les forêts sèches de Madagascar, Vol. 1 (pp. 76–105). Malagasy Nature.

[ece37224-bib-0107] Rakotoarinivo, M. , Blach‐Overgaard, A. , Baker, W. J. , Dransfield, J. , Moat, J. , & Svenning, J.‐C. (2013). Palaeo‐precipitation is a major determinant of palm species richness patterns across Madagascar: A tropical biodiversity hotspot. Proceedings of the Royal Society B: Biological Sciences, 280(1757), 20123048. 10.1098/rspb.2012.3048 PMC3619483 23427173

[ece37224-bib-0108] Ramírez‐Barrera, S. M. , Velasco, J. A. , Orozco‐Téllez, T. M. , Vázquez‐López, A. M. , & Hernández‐Baños, B. E. (2019). What drives genetic and phenotypic divergence in the Red‐crowned Ant tanager (*Habia rubica*, Aves: Cardinalidae), a polytypic species? Ecology and Evolution, 9(21), 12339–12352. 10.1002/ece3.5742 31832165 PMC6854386

[ece37224-bib-0109] Randrianarivony, T. N. , Ramarosandratana, A. V. , Andriamihajarivo, T. H. , Rakotoarivony, F. , Jeannoda, V. H. , Randrianasolo, A. , & Bussmann, R. W. (2017). The most used medicinal plants by communities in Mahaboboka, Amboronabo, Mikoboka, Southwestern Madagascar. Journal of Ethnobiology and Ethnomedicine, 13(1), 19. 10.1186/s13002-017-0147-x 28279184 PMC5345199

[ece37224-bib-0110] Rasmussen, H. N. (2002). Recent developments in the study of orchid mycorrhiza. Plant and Soil, 244(1), 149–163. 10.1023/A:1020246715436

[ece37224-bib-0111] Rasmussen, H. N. , Dixon, K. W. , Jersáková, J. , & Těšitelová, T. (2015). Germination and seedling establishment in orchids: A complex of requirements. Annals of Botany, 116(3), 391–402. 10.1093/aob/mcv087 26271118 PMC4549959

[ece37224-bib-0112] Raymond, M. , & Rousset, F. (1995). GENEPOP (version 1.2): Population genetics software for exact tests and ecumenicism. Journal of Heredity, 86, 248–249. 10.1093/oxfordjournals.jhered.a111573

[ece37224-bib-0113] Razafinarivo, N. J. , Guyot, R. , Davis, A. P. , Couturon, E. , Hamon, S. , Crouzillat, D. , Rigoreau, M. , Dubreuil‐Tranchant, C. , Poncet, V. , De Kochko, A. , Rakotomalala, J.‐J. , & Hamon, P. (2013). Genetic structure and diversity of coffee (*Coffea*) across Africa and the Indian Ocean islands revealed using microsatellites. Annals of Botany, 111(2), 229–248. 10.1093/aob/mcs283 23275631 PMC3555535

[ece37224-bib-0114] Ribeiro, E. D. C. , Batjes, N. H. , & van Oostrum, A. J. M. (2018). World Soil Information Service (WoSIS) ‐ Towards the standardization and harmonization of world soil data: Procedures Manual 2018 (No. 2018/01). ISRIC ‐ World Soil Information. 10.17027/ISRIC-WDCSOILS.20180001

[ece37224-bib-0115] Ronfort, J. , Jenczewski, E. , & Muller, M. H. (2005). Les flux de gènes et leur impact sur la structure de la diversité génétique. Le cas des prairies: Génétique et prairies. Fourrages (Versailles), 182, 275–286.

[ece37224-bib-0116] Rosenberg, N. A. , Burke, T. , Elo, K. , Feldman, M. W. , Freidlin, P. J. , Groenen, M. A. M. , & Weigend, S. (2001). Empirical Evaluation of Genetic Clustering Methods Using Multilocus Genotypes From 20 Chicken Breeds. Genetics, 159(2), 699–713. 11606545 10.1093/genetics/159.2.699 PMC1461842

[ece37224-bib-0117] Rousset, F. (2008). genepop’007: A complete re‐implementation of the genepop software for Windows and Linux. Molecular Ecology Resources, 8(1), 103–106. 10.1111/j.1471-8286.2007.01931.x 21585727

[ece37224-bib-0118] Rundle, H. D. , & Nosil, P. (2005). Ecological speciation. Ecology Letters, 8(3), 336–352. 10.1111/j.1461-0248.2004.00715.x

[ece37224-bib-0119] Schmid, M. , & Guillaume, F. (2017). The role of phenotypic plasticity on population differentiation. Heredity, 119(4), 214–225. 10.1038/hdy.2017.36 28745716 PMC5597782

[ece37224-bib-0120] Shafer, A. B. A. , & Wolf, J. B. W. (2013). Widespread evidence for incipient ecological speciation: A meta‐analysis of isolation‐by‐ecology. Ecology Letters, 16(7), 940–950. 10.1111/ele.12120 23627762

[ece37224-bib-0121] Slatkin, M. (2008). Linkage disequilibrium — understanding the evolutionary past and mapping the medical future. Nature Reviews Genetics, 9(6), 477–485. 10.1038/nrg2361 PMC5124487 18427557

[ece37224-bib-0122] Sobel, J. M. (2016). Speciation, Geography of. Encyclopedia of Evolutionary Biology, 4, 183–191. 10.1016/B978-0-12-800049-6.00333-4

[ece37224-bib-0123] Sobel, J. M. , Chen, G. F. , Watt, L. R. , & Schemske, D. W. (2009). The biology of speciation. Evolution, 64(2), 295–315. 10.1111/j.1558-5646.2009.00877.x 19891628

[ece37224-bib-0124] Soliva, M. , & Widmer, A. (2003). Gene flow across species boundaries in sympatric, sexually deceptive *Ophrys* (orchidaceae) Species. Evolution, 57(10), 2252–2261. 10.1111/j.0014-3820.2003.tb00237.x 14628913

[ece37224-bib-0125] Soto‐Arenas, M. Á. , & Cribb, P. (2010). A new infrageneric classification and synopsis of the genus *Vanilla* Plum. ex mill. (Orchidaceae: Vanillinae). Lankesteriana, 9(3), 355–398. 10.15517/lank.v0i0.12071

[ece37224-bib-0126] Stone, J. L. , Crystal, P. A. , Devlin, E. E. , Downer, R. H. L. , & Cameron, D. S. (2012). Highest genetic diversity at the northern range limit of the rare orchid *Isotria medeoloides* . Heredity, 109(4), 215–221. 10.1038/hdy.2012.31 22692268 PMC3464020

[ece37224-bib-0127] Sun, M. , Schlüter, P. M. , Gross, K. , & Schiestl, F. P. (2015). Floral isolation is the major reproductive barrier between a pair of rewarding orchid sister species. Journal of Evolutionary Biology, 28(1), 117–129. 10.1111/jeb.12544 25382492

[ece37224-bib-0128] Svenning, J.‐C. (2003). Deterministic Plio‐Pleistocene extinctions in the European cool‐temperate tree flora. Ecology Letters, 6(7), 646–653. 10.1046/j.1461-0248.2003.00477.x

[ece37224-bib-0129] Swarts, N. D. , Sinclair, E. A. , Krauss, S. L. , & Dixon, K. W. (2009). Genetic diversity in fragmented populations of the critically endangered spider orchid *Caladenia huegelii*: Implications for conservation. Conservation Genetics, 10(5), 1199–1208. 10.1007/s10592-008-9651-9

[ece37224-bib-0130] Tremblay, R. L. (1992). Trends in the pollination ecology of the Orchidaceae: Evolution and systematics. Canadian Journal of Botany, 70(3), 642–650. 10.1139/b92-083

[ece37224-bib-0131] van Aarle, I. M. , Olsson, P. A. , & Soderstrom, B. (2002). Arbuscular mycorrhizal fungi respond to the substrate pH of their extraradical mycelium by altered growth and root colonization. New Phytologist, 155(1), 173–182. 10.1046/j.1469-8137.2002.00439.x 33873298

[ece37224-bib-0132] Van der Niet, T. , Peakall, R. , & Johnson, S. D. (2014). Pollinator‐driven ecological speciation in plants: New evidence and future perspectives. Annals of Botany, 113(2), 199–212. 10.1093/aob/mct290 24418954 PMC3890394

[ece37224-bib-0133] Vences, M. , Wollenberg, K. C. , Vieites, D. R. , & Lees, D. C. (2009). Madagascar as a model region of species diversification. Trends in Ecology & Evolution, 24(8), 456–465. 10.1016/j.tree.2009.03.011 19500874

[ece37224-bib-0134] Waeber, P. O. , Wilm, L. , Ramamonjisoa, B. , Garcia, C. , Rakotomalala, D. , Rabemananjara, Z. H. , Kull, C. A. , Ganzhorn, J. U. , & Sorg, J.‐P. (2015). Dry forests in Madagascar: Neglected and under pressure. International Forestry Review, 17(2), 127–148. 10.1505/146554815815834822

[ece37224-bib-0135] Wahlund, S. (1928). Zusammensetzung Von Populationen Und Korrelationserscheinungen Vom Standpunkt Der Vererbungslehre Aus Betrachtet. Hereditas, 11(1), 65–106. 10.1111/j.1601-5223.1928.tb02483.x

[ece37224-bib-0136] Wang, I. J. (2013). Examining the full effects of landscape heterogeneity on spatial genetic variation: a multiple matrix regression approach for quantifying geographic and ecological isolation. Evolution, 67(12), 3403–3411. 10.1111/evo.12134 24299396

[ece37224-bib-0137] Wang, I. J. , & Bradburd, G. S. (2014). Isolation by environment. Molecular Ecology, 23(23), 5649–5662. 10.1111/mec.12938 25256562

[ece37224-bib-0138] Wang, J. (2017). The computer program structure for assigning individuals to populations: Easy to use but easier to misuse. Molecular Ecology Resources, 17(5), 981–990. 10.1111/1755-0998.12650 28028941

[ece37224-bib-0139] Waples, R. S. (2006). A bias correction for estimates of effective population size based on linkage disequilibrium at unlinked gene loci*. Conservation Genetics, 7(2), 167–184. 10.1007/s10592-005-9100-y

[ece37224-bib-0140] Waples, R. S. (2015). Testing for Hardy‐Weinberg Proportions: Have We Lost the Plot? Journal of Heredity, 106(1), 1–19. 10.1093/jhered/esu062 25425676

[ece37224-bib-0141] Waples, R. S. (2018). Null Alleles and FIS × FST Correlations. Journal of Heredity, 109(4), 457–461. 10.1093/jhered/esy013 29554281

[ece37224-bib-0142] Warren, B. H. , Simberloff, D. , Ricklefs, R. E. , Aguilée, R. , Condamine, F. L. , Gravel, D. , Morlon, H. , Mouquet, N. , Rosindell, J. , Casquet, J. , Conti, E. , Cornuault, J. , Fernández‐Palacios, J. M. , Hengl, T. , Norder, S. J. , Rijsdijk, K. F. , Sanmartín, I. , Strasberg, D. , Triantis, K. A. , … Thébaud, C. (2015). Islands as model systems in ecology and evolution: Prospects fifty years after MacArthur‐Wilson. Ecology Letters, 18(2), 200–217. 10.1111/ele.12398 25560682

[ece37224-bib-0143] Waser, N. M. , & Campbell, D. R. (2004). Ecological speciation in flowering plants. In U. Dieckmann , M. Doebeli , J. A. J. Metz , & D. Tautz (Eds.), Adaptive speciation (pp. 264–277). Cambridge University Press.

[ece37224-bib-0144] Weiner, J. (2019). PCA 3D: Getting PCA plots quickly. Retrieved from https://CRAN.R‐project.org/package=pca3d

[ece37224-bib-0145] Weir, B. S. , & Cockerham, C. C. (1984). Estimating F‐statistics for the analysis of population structure. Evolution, 38(6), 1358–1370. 28563791 10.1111/j.1558-5646.1984.tb05657.x

[ece37224-bib-0146] Wilmé, L. , Goodman, S. M. , & Ganzhorn, J. U. (2006). Biogeographic evolution of Madagascar’s microendemic biota. Science, 312(5776), 1063–1065. 10.1126/science.1122806 16709785

[ece37224-bib-0147] Winn, A. A. , & Gross, K. L. (1993). Latitudinal variation in seed weight and flower number in *Prunella vulgaris* . Oecologia, 93(1), 55–62. 10.1007/BF00321191 28313774

[ece37224-bib-0148] Wright, S. (1931). Evolution in Mendelian Populations. Genetics, 16(2), 97–159. 10.1093/genetics/16.2.97 17246615 PMC1201091

[ece37224-bib-0149] Wright, S. (1943). Isolation by Distance. Genetics, 28(2), 114–138. 17247074 10.1093/genetics/28.2.114 PMC1209196

[ece37224-bib-0150] Yokoya, K. , Zettler, L. W. , Kendon, J. P. , Bidartondo, M. I. , Stice, A. L. , Skarha, S. , Corey, L. L. , Knight, A. C. , & Sarasan, V. (2015). Preliminary findings on identification of mycorrhizal fungi from diverse orchids in the Central Highlands of Madagascar. Mycorrhiza, 25(8), 611–625. 10.1007/s00572-015-0635-6 25771863

[ece37224-bib-0151] Zhang, H.‐X. , Zhang, M.‐L. , & Williams, D. M. (2014). Genetic evidence and species distribution modelling reveal the response of *Larix sibirica* and its related species to Quaternary climatic and ancient historical events. Biochemical Systematics and Ecology, 54, 316–325. 10.1016/j.bse.2014.02.017

