# Supplementary material for: Drivers of population divergence and species differentiation in a recent group of indigenous orchids (Vanilla spp.) in Madagascar
Source: Ecol Evol. 2021 Feb 24;11(6):2681–700. doi: 10.1002/ece3.7224 (PMC7981232; doi:10.1002/ece3.7224)

Suppplementary material

Drivers of population divergence and species differentiation in a recent group of indigenous orchids (*Vanilla spp.*) in Madagascar (Andriamihaja et al.)

Table S1: Geographical location, elevation and number of genotyped individuals (n) for the studied populations.

Sites	Code	Longitude	Latitude	Elevation (m)	n
Montagne des Français	MDF	49.354203	-12.335875	215	29
Analamerana	ANM	49.477491	-12.724982	58	27
Ankarana	ANK	49.12918	-12.952675	117	22
Ambanja	AMB	48.50000	-13.610000	143	27
Maromandia	MND	48.088309	-14.218457	41	41
Befandrama	BFD	47.760048	-14.852435	132	24
Antsianitia	ATS	46.427925	-15.570435	19	27
Marosely	MSL	47.578808	-15.648803	280	41
Complexe Mahavavy-Kinkony	CMK	45.824605	-15.945627	16	26
Betaramahamay	BTM	47.444201	-15.964932	211	34
Baie de Baly	BBL	45.273094	-16.068887	15	26
Ankarafantsika	AKF	46.816657	-16.302281	95	24
Namoroka	NMK	45.302633	-16.402629	107	24
Kirindy Forest	KRD	44.657645	-20.067082	55	33
Kirindy Mitea	KRM	44.045367	-20.820437	24	29
Ankililoaka	AKL	43.777311	-22.791984	274	25
Zombitse Vohibasia	ZVB	44.675825	-22.911742	749	17
Antandroka	ATD	43.518649	-22.946485	31	23
Anja	ANJ	43.605442	-23.085189	11	24
Mahaleotse	MLT	44.095938	-23.518483	89	38
Saint-Augustin	SAG	43.780511	-23.538178	20	9
Manompana	MAN	49.610000	-16.960000	41	11
Andringitra	AND	46.777304	-22.08464	887	30

Table S2: Characteristics of the seven nuclear microsatellites of the genus *Vanilla* developed by Gigant et al. (2011) from *V. humblotii* and *V. roscheri*.

Locus name	Repeat motif	Primer sequence (5′-3′)	GenBank
mVroCIR04	(ga) 18	F: AAGGTACAAGATCCCCGTCA<br>R: CCGCGAGCTTTATTCTACCA	JN222574
mVroCIR05	(ga) 15	F: GCTATTTCCCACGAACCCTA<br>R: AACCATTGCCAGAAGCCTAA	JN222575
mVhuCIR03	(ag) 24	F: GTCGCATTCACATAGCTTCG<br>R: TCTTCCTACCGCTGTCGTCT	JN222562
mVhuCIR04	(ag) 13	F: GGATACTTCCGGTGACTCCA<br>R: GCTCTGGCTCTGTGGTTAGG	JN222563
mVhuCIR06	(tg) 10	F: CTTGGTTGTTCTGCTGGATG<br>R: CCCTAGAACCCATGTCTTGC	JN222564
mVhuCIR07	(ga) 16	F: CCACGTAGATCAAACACAGCA<br>R: AAGAAACTGTTAGAAATCCCAAGC	JN222565
mVhuCIR08	(ga) 15	F: TTAACTCTTCGCCCATTAAGC<br>R: CCTTCGAGCTTTCCGTTC	JN222566

Table S3: Mean values of the environmental characteristics of the 23 leafless *Vanilla* populations in Madagascar. For population codes, see table S1. For variable codes and units, see table S4.

	MDF	ANM	ANK	AMB	MND	BFD	ATS	MSL	CMK	BTM	BBL	AKF	NMK	MAN	KRD	KRM	AND	AKL	ZVB	ATD	ANJ	MLT	SAG
**Elv (m)**	216	58	117	16	50	132	19	283	16	211	15	96	107	20	55	25	942	274	749	28	11	89	22
**NDVIM**	0.68	0.67	0.68	0.79	0.62	0.55	0.51	0.61	0.62	0.53	0.69	0.65	0.62	0.84	0.72	0.67	0.58	0.56	0.49	0.63	0.62	0.53	0.56
**pH**	5.89	6.04	5.63	5.73	5.65	6.22	6.14	5.72	5.84	5.81	6.25	5.63	5.82	5.3	5.86	6.1	5.6	6.38	5.68	7.3	6.73	6.53	6.78
**Sand (%)**	60	65	63	53	57	69	55	65	69	66	65	59	66	49	67	65	54	61	61	72	64	61	71
**Clay (%)**	23	21	18	26	24	17	25	19	17	16	19	22	19	28	22	23	27	24	23	18	22	24	20
**Silt (%)**	17	14	18	21	19	15	20	16	13	15	15	19	15	23	12	12	18	15	16	9	13	16	9
**BIO1**	25	26	26	26	26	26	26	25	27	26	27	26	26	24	25	25	20	24	22	25	25	24	24
**BIO2**	9	10	10	10	11	12	11	13	12	14	10	14	12	9	13	12	12	13	14	12	12	14	11
**BIO3**	74	75	13	70	71	78	70	76	71	76	66	81	73	62	63	60	65	63	67	61	58	62	58
**BIO4**	117	131	136	145	142	113	147	152	144	167	146	121	136	214	221	250	246	263	264	276	284	310	292
**BIO5**	31	32	32	33	34	33	34	33	35	35	34	35	34	32	35	35	29	34	33	34	34	35	34
**BIO6**	19	19	19	19	18	19	18	16	18	16	19	18	18	17	15	15	10	13	11	15	15	13	15
**BIO7**	12	14	13	14	16	15	16	17	17	18	15	17	16	14	21	20	19	21	21	20	20	22	19
**BIO8**	26	27	27	27	27	27	28	26	28	27	28	27	27	26	27	28	23	26	25	28	28	28	27
**BIO9**	24	25	24	24	24	25	26	23	25	24	25	25	24	23	22	22	17	20	19	22	21	21	21
**BIO10**	26	27	27	27	27	27	28	26	28	28	28	27	27	23	27	28	23	26	25	28	28	28	28
**BIO11**	24	24	24	24	24	25	24	23	25	24	25	25	24	16	22	22	17	20	19	21	21	20	21
**BIO12**	1139	1390	1563	2064	1797	1552	1683	1490	1446	1473	1228	1440	1325	3316	919	703	963	664	742	577	575	604	543
**BIO13**	305	322	403	497	479	452	411	458	418	444	403	421	405	332	276	214	246	167	188	144	146	156	137
**BIO14**	11	27	14	27	8	2	24	0	12	0	1	1	2	104	2	1	6	3	5	2	3	4	3
**BIO15**	117	94	114	100	110	124	94	132	117	129	135	125	127	43	127	131	110	112	107	113	112	109	109
**BIO16**	773	825	1035	1264	1154	1084	993	1096	988	1078	935	1013	958	1277	663	532	626	450	482	399	394	404	365
**BIO17**	47	97	45	84	29	8	105	2	42	2	6	6	9	370	6	6	28	11	19	11	13	16	14
**BIO18**	520	825	873	880	764	826	699	838	707	674	572	954	289	1247	663	532	626	450	482	399	350	349	321
**BIO19**	55	100	46	90	29	13	130	2	42	2	6	6	9	603	6	6	34	13	20	12	14	18	17

Table S4: List of the 25 environmental variables

Code	Variables	Units
Elv (m)	Elevation	meter
NDVIM	Maximum Normalized Difference Vegetation index	
pH	Soil pH in H20 at 5 cm of depth	
Sand	Quantity of sand between 5 and 15 cm of deep	%
Clay	Quantity of clay at 5 cm of depth	%
Silt	Quantity of silt at 5 cm of depth	%
BIO1	Annual Mean Temperature	°C
BIO2	Mean diurnal range (mean of monthly (max temp-min temp)	°C
BIO3	Isothermality	BIO2/BIO7 * 100
BIO4	Temperature seasonality	Standard deviation * 100
BIO5	Max temperature of warmest month	°C
BIO6	Min temperature of coldest month	°C
BIO7	Temperature annual range (BIO5-BIO6)	°C
BIO8	Mean temperature of wettest quarter	°C
BIO9	Mean temperature of driest quarter	°C
BIO10	Mean temperature of warmest quarter	°C
BIO11	Mean temperature of coldest quarter	°C
BIO12	Annual precipitation	millimeter
BIO13	Precipitation of wettest month	millimeter
BIO14	Precipitation of driest month	millimeter
BIO15	Precipitation seasonality	Coefficient of variation * 100
BIO16	Precipitation of wettest quarter	millimeter
BIO17	Precipitation of driest quarter	millimeter
BIO18	Precipitation of warmest quarter	millimeter
BIO19	Precipitation of coldest quarter	millimeter

Table S5: Matrix of pairwise FST values between 23 leafless *Vanilla* population from Madagascar calculated from neutral microsatellite datasets, all values are significant (p-value <0.001). For population code, see Table S1.

Pop	MDF	ANM	ANK	AMB	MND	BFD	ATS	MSL	CMK	BTM	BBL	AKF	NMK	KRD	KRM	AKLL	ZVB	ATD	ANJ	MLT	SAG	MAN
ANM	0.30																					
ANK	0.30	0.04																				
AMB	0.31	0.05	0.09																			
MND	0.29	0.03	0.08	0.01																		
BFD	0.19	0.17	0.19	0.21	0.17																	
ATS	0.21	0.11	0.14	0.16	0.14	0.12																
MSL	0.21	0.12	0.14	0.17	0.14	0.11	0.03															
CMK	0.22	0.04	0.07	0.07	0.05	0.11	0.03	0.04														
BTM	0.22	0.21	0.22	0.26	0.22	0.03	0.15	0.14	0.14													
BBL	0.21	0.10	0.12	0.15	0.12	0.09	0.02	0.01	0.02	0.11												
AKF	0.22	0.13	0.16	0.19	0.16	0.11	0.03	0.02	0.04	0.14	0.02											
NMK	0.23	0.13	0.16	0.19	0.15	0.12	0.04	0.01	0.05	0.15	0.02	0.01										
KRD	0.20	0.12	0.15	0.17	0.14	0.12	0.02	0.02	0.04	0.15	0.03	0.01	0.02									
KRM	0.38	0.27	0.28	0.31	0.28	0.27	0.24	0.20	0.22	0.28	0.22	0.22	0.24	0.18								
AKLL	0.19	0.17	0.19	0.22	0.18	0.07	0.09	0.09	0.09	0.10	0.06	0.09	0.11	0.09	0.28							
ZVB	0.24	0.15	0.18	0.22	0.17	0.14	0.06	0.02	0.06	0.16	0.03	0.03	0.02	0.02	0.24	0.09						
ATD	0.38	0.30	0.31	0.34	0.30	0.27	0.25	0.22	0.25	0.29	0.24	0.24	0.24	0.19	0.07	0.29	0.25					
ANJ	0.46	0.38	0.39	0.42	0.37	0.37	0.35	0.30	0.33	0.37	0.32	0.33	0.34	0.28	0.07	0.38	0.35	0.12				
MLT	0.33	0.23	0.24	0.27	0.24	0.21	0.21	0.16	0.19	0.22	0.17	0.17	0.19	0.16	0.12	0.23	0.20	0.17	0.17			
SAG	0.40	0.31	0.33	0.35	0.31	0.28	0.28	0.23	0.26	0.28	0.25	0.24	0.27	0.22	0.16	0.31	0.28	0.20	0.20	0.06		
MAN	0.32	0.29	0.30	0.32	0.28	0.22	0.14	0.17	0.20	0.23	0.19	0.19	0.21	0.19	0.39	0.23	0.24	0.41	0.52	0.33	0.44	
AND	0.37	0.37	0.39	0.41	0.35	0.30	0.25	0.24	0.26	0.30	0.25	0.25	0.27	0.23	0.46	0.28	0.28	0.49	0.60	0.39	0.53	0.41

Figure S1: Plot of (a) the mean posterior probabilities of K, Ln P(X|K), as implemented in STRUCTURE Software (Pritchard et al.,2000) and (b) the distribution ΔK values from STRUCTURE with K=4 based on Evanno method (Evanno et al.,2005).

(a)

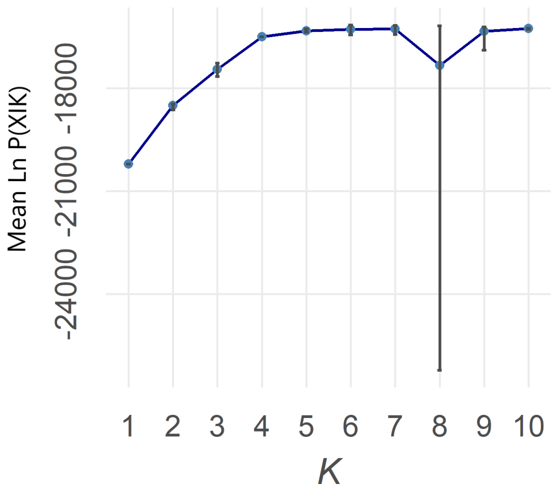

(b)

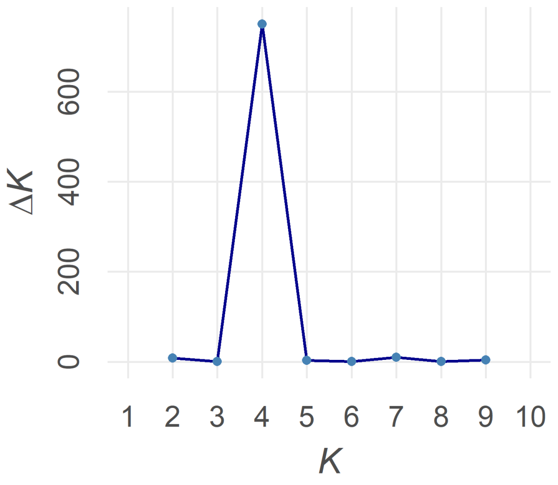

Figure S2: Contribution (%) of the 26 morphological variables to the three principal components of PCA: (a): PC1, (b): PC2, (c): PC3

(a)

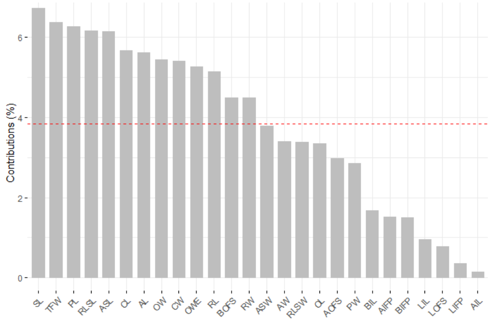

(b)

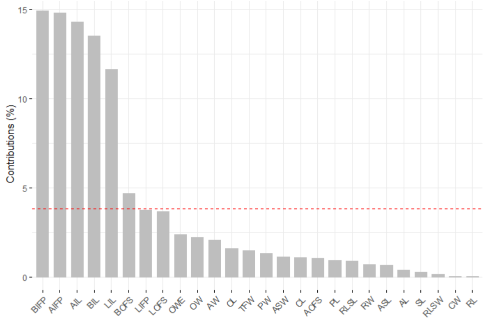

(c)

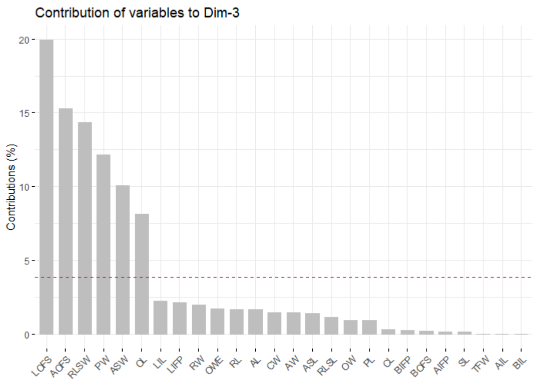

Figure S3: 3D PCA plot of 14 uncorrelated environmental variables of 23 populations (a) and contribution (%) of the 14 environmental variables to the three principal components of PCA: (a): PC1, (b): PC2, (c): PC3

(**a**)

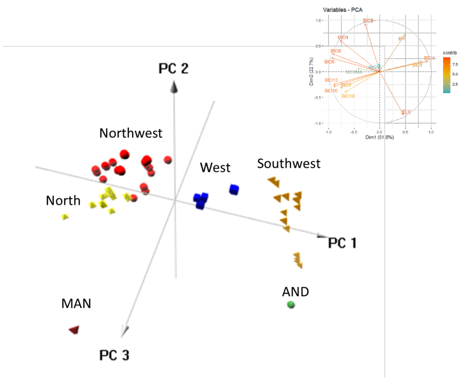

(**b**)

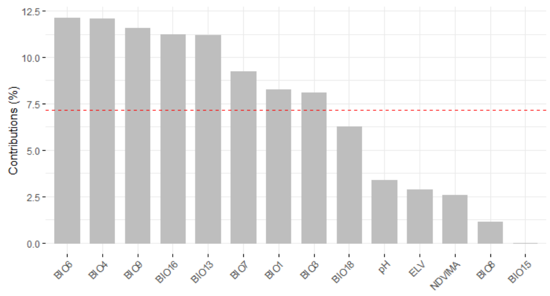

(c)

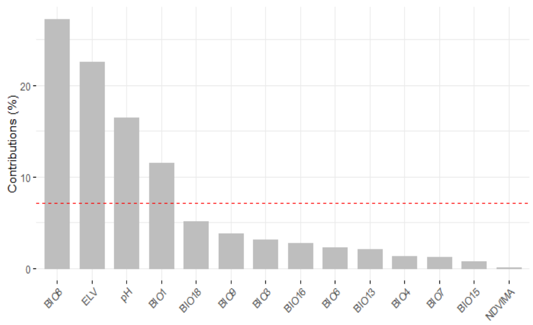

(d)

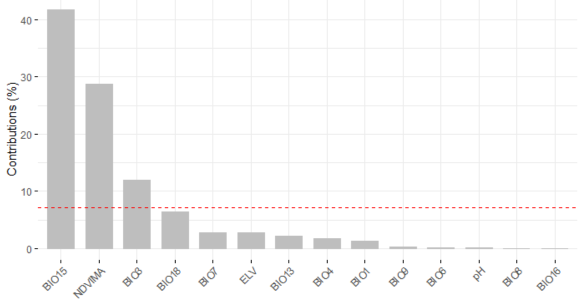

Figure S4: Contribution (%) of the 14 morphological variables to the three principal components of PCA: (a): PC1, (b): PC2, (c): PC3

(**a**)

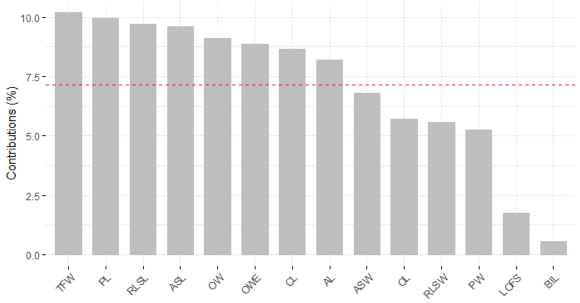

(**b**)

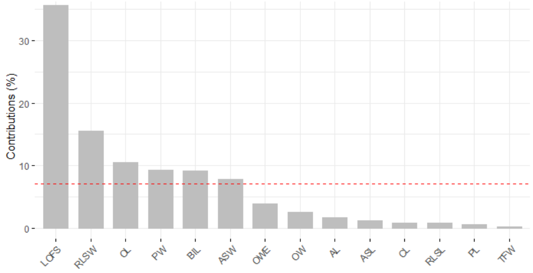

(c)

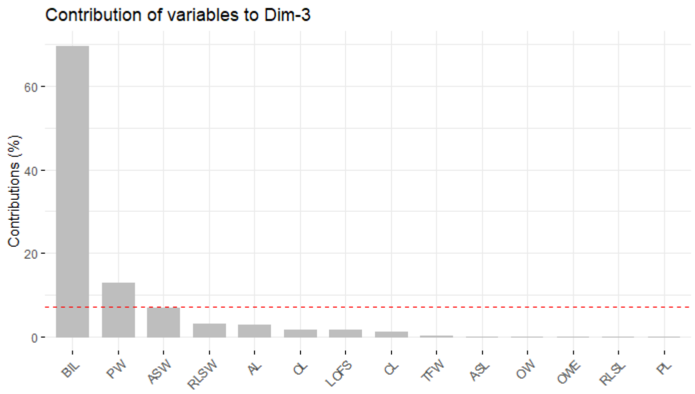

Figure S5: Contribution (%) of the 9 environmental variables to the three principal components of PCA: (a): PC1, (b): PC2, (c): PC3

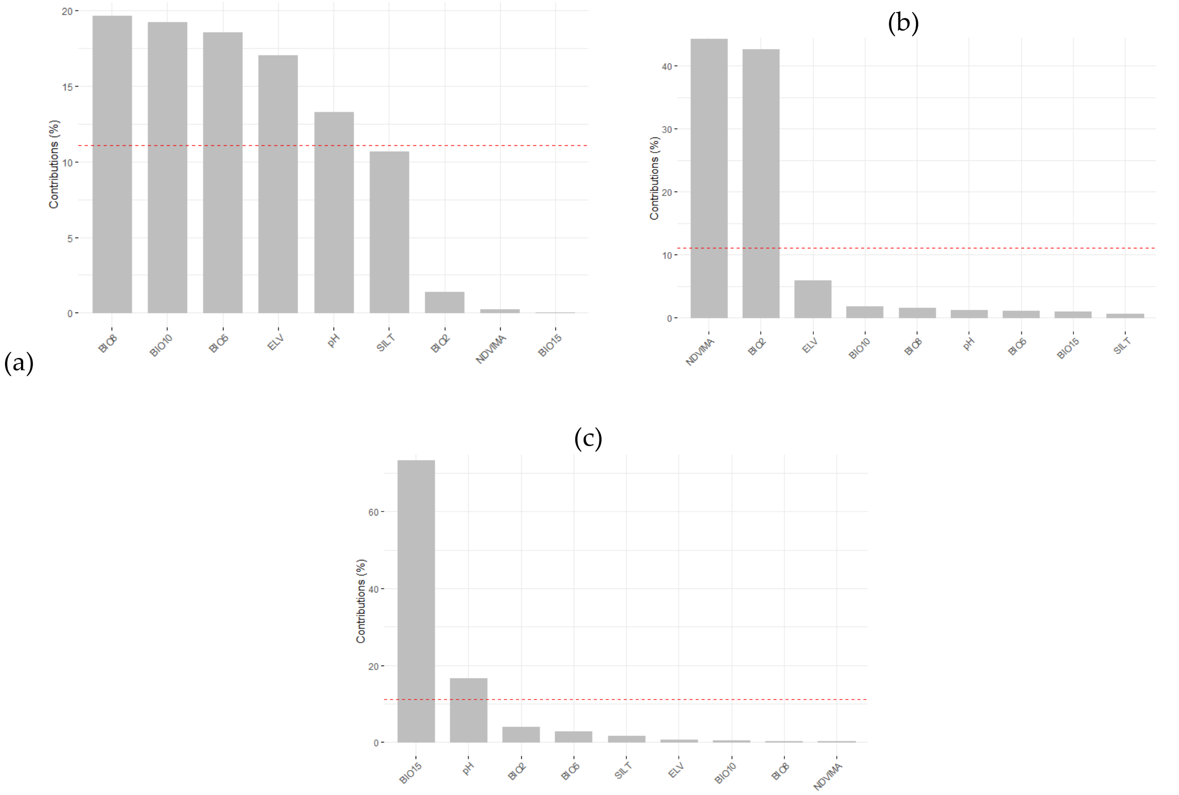

Supplement: Supplementary file 1 — Appendix S1 [file ECE3-11-2681-s001.pdf]
